# Comprehensive Review of Antiplatelet Therapy in Secondary Prevention of Myocardial Infarction: Mechanisms, Clinical Evidence, and Future Directions

**DOI:** 10.1155/cdr/3961611

**Published:** 2026-06-01

**Authors:** May Thu Kyaw, Arkar Moe

**Affiliations:** ^1^ Internal Medicine, University of Medicine 2, Yangon, Myanmar; ^2^ Heart and Vascular Centre, Victoria Hospital, Yangon, Myanmar, lhsc.on.ca

**Keywords:** antiplatelet therapy, clinical guidelines, high on-treatment platelet reactivity, myocardial infarction, P2Y12 inhibitors, risk stratification, secondary prevention

## Abstract

Atherosclerotic cardiovascular disease (ASCVD), particularly myocardial infarction (MI), is the leading cause of mortality and morbidity worldwide. The pathophysiology of MI involves atherosclerotic plaque rupture with thrombus formation, interrupting myocardial blood supply with subsequent myocardial necrosis. Despite advances in primary prevention and acute revascularization strategies for MI, recurrent ischemic events and stent thrombosis continue to pose challenging issues for clinicians. Consequently, effective secondary prevention, specifically antiplatelet therapy (aspirin and P2Y12 inhibitors), is the cornerstone of the prevention of recurrent ischemia and cardiovascular death to improve long‐term prognosis. However, the optimal choice and duration of antiplatelet therapy should be individualized depending on risk factors and comorbidities. This review will explore the mechanisms of thrombosis in MI, the role of antiplatelet therapy in secondary prevention after MI, and the clinical evidence supporting various antiplatelet agents, focusing on their pharmacological profiles, efficacy, safety considerations, guideline recommendations, and application in special populations based on available clinical trial data while highlighting key controversies, knowledge gaps, and emerging strategies such as biomarker‐guided and genotype‐guided therapy.

## 1. Introduction

Globally, ischemic heart disease (IHD) affects approximately 1.72% of the world’s population, and myocardial infarction (MI) remains the leading cause of death, accounting for 9 million fatalities annually [1]. The incidence of MI is influenced by geographic, socioeconomic, lifestyle, genetic predisposition, and healthcare factors. Despite advanced acute revascularization strategies and thrombolysis, the mortality rate of acute myocardial infarction (AMI) remains significant depending on the severity and location of MI and timely intervention, ranging from 19% to 84%, as reported by the REGICOR study [2]. Higher mortality rates are observed in older patients with multiple comorbid conditions. Although modern medical and interventional strategies have significantly reduced immediate mortality from AMI, the overall disease burden persists, largely due to the sequelae of MI, such as heart failure, arrhythmias, and recurrent ischemic events. Post‐MI, cardiovascular mortality is approximately 10% within the first year, with an ongoing annual death rate of around 5%, which persists indefinitely. Cumulatively, the cardiovascular mortality rate after 15 years approaches 70% [3]. According to 12‐year national data from the United States, the total annual healthcare costs related to AMI in 2016 were estimated at $84.9 billion, encompassing both direct expenses, such as hospitalizations, interventions, and medications, and indirect costs from lost productivity due to morbidity and long‐term disability [4].

## 2. Discussion

### 2.1. Risk Factors and Pathophysiology of Thrombosis in AMI

Risk factors for IHD include smoking, diabetes mellitus (DM), hypertension, dyslipidemia, chronic kidney disease (CKD), obesity, advanced age, family history, and genetic predisposition. The pathogenesis of thrombosis in MI is multifactorial and primarily driven by atherosclerosis, a chronic inflammatory process characterized by lipid accumulation, endothelial dysfunction, and plaque formation within the coronary arteries. The rupture or erosion of vulnerable atherosclerotic plaques—composed of large lipid cores covered by thin fibrous caps—exposes prothrombotic elements such as collagen and tissue factor to the circulating blood [5]. This exposure activates glycoprotein receptors on platelets, leading to platelet adhesion, aggregation, and degranulation mediated by pathways involving adenosine diphosphate (ADP) and thromboxane A2 (TXA2) [6]. Subsequently, the exposure of tissue factor from unstable plaques initiates the extrinsic coagulation cascade, resulting in thrombin generation and fibrin formation [7]. The culmination of these processes is intracoronary thrombus formation, which impairs coronary blood flow and induces myocardial ischemia. An understanding of this pathophysiological sequence underscores the importance of antiplatelet therapies targeting TXA2 synthesis and ADP‐mediated P2Y12 receptor signaling pathways, as these interventions can reduce thrombus propagation and the risk of MI and recurrent ischemic events.

### 2.2. Risk of Recurrent Ischemic Events

Despite advances in acute revascularization and pharmacological therapies, survivors of MI continue to face significant cardiovascular morbidities, including left ventricular systolic and diastolic dysfunction, arrhythmias, and recurrent ischemic events. A study from the United States revealed that the 5‐year rate of major adverse cardiovascular events (MACEs), including recurrent MI, ischemic stroke, and cardiovascular death, was 33.4%, with most events occurring within the first year after discharge [8]. Additionally, an analysis of the international Global Registry of Acute Coronary Events (GRACE) registry for acute coronary syndrome (ACS) found that the 5‐year mortality rate following MI was approximately 20%, with more than two‐thirds of these deaths occurring within 30 days after hospital discharge [9]. High rates of recurrent cardiovascular events were observed in the older population, particularly among those with CKD and diabetes. Patients with AMI have a high risk of recurrent ischemia due to ongoing proinflammatory states, persistent vulnerable plaques, and associated metabolic conditions such as diabetes, hypertension, and dyslipidemia, as well as smoking and inadequate secondary prevention treatments. The main causes of reinfarction were stent thrombosis and in‐stent restenosis, accounting for approximately one‐quarter of cases, followed by disease progression at 12% and persistent coronary artery disease (CAD) at 11% [10]. Recurrent MI has a high 1‐year mortality of approximately 38% [11]. According to the REACH registry, after a 4‐year follow‐up, patients with a previous MI exhibited a high residual ischemic risk that increased annually—from 4.7% during the first year to a cumulative rate of 15.1% over 4 years [12].

### 2.3. Role of Antiplatelets in Secondary Prevention

While primary prevention strategies such as blood pressure and diabetes control, lipid lowering, smoking cessation, and lifestyle modifications are important, these alone are not sufficient to reduce recurrent ischemic events after established cardiovascular disease (CVD). Given the central role of antiplatelet agents in the inhibition of key receptors and enzymes involved in platelet aggregation and coagulation, they are fundamental in secondary prevention after MI to reduce MACEs and improve prognosis, especially when combined with statins, beta‐blockers, and angiotensin‐converting enzyme inhibitors (ACEIs). Antiplatelet therapy plays an integral role in secondary prevention of MACEs, especially after percutaneous coronary intervention (PCI). A pooled analysis of 11,219 patients demonstrated that premature discontinuation of dual antiplatelet therapy (DAPT) following drug‐eluting stent (DES) implantation is strongly associated with an increased risk of stent thrombosis [13]. Moreover, the ADAPT‐DES registry highlighted the importance of adequate P2Y12 inhibition after MI, especially within the first 30 days after stent implantation [14]. However, while these studies establish the necessity of antiplatelet therapy, they also reveal the complexity of balancing ischemic protection against bleeding risk—a tension that has driven the evolution toward shorter DAPT durations and de‐escalation strategies, particularly in higher‐risk populations.

In recent decades, antiplatelet agents like aspirin and P2Y12 inhibitors have been developed to target different steps in the platelet activation process. Many randomized clinical trials have proved that these antiplatelet agents significantly reduce the risk of recurrent MI and stroke, ultimately improving survival rates. The most widely used antiplatelets are aspirin, dipyridamole, cilostazol, and P2Y12 inhibitors (clopidogrel, ticagrelor, and prasugrel). Following an index ACS, after a certain duration of DAPT, de‐escalation to single antiplatelet therapy—particularly with a potent P2Y12 inhibitor—has gained increasing popularity to help mitigate bleeding risk. Moreover, a shorter duration of DAPT has become more widely adopted, particularly in patients with advanced age and high bleeding risk (HBR). The 2012 RESET and 2013 OPTIMIZE trials demonstrated the efficacy and safety of shortened DAPT following DES implantation. The RESET trial evaluated whether 3 months of DAPT was noninferior to 12 months of DAPT in preventing MACEs at 1 year, with a primary composite endpoint occurring in 4.7% of both groups (*p* = 0.84) [15]. Likewise, the OPTIMIZE trial confirmed that 3 months of DAPT was noninferior to 12 months of DAPT regarding net adverse clinical and cerebral events at 12 months, with rates of 6.0% versus 5.8%, respectively (*p* = 0.002) [16]. However, the optimal selection and duration of antiplatelet therapy are individualized and challenging, as this approach requires careful judgment between the benefits of preventing ischemia and the risk of bleeding. Critically, the generalizability of these shorter DAPT trials is limited by their enrollment of predominantly low‐risk patients with simple lesions, raising questions about the applicability of these findings to complex PCI or patients with multiple high‐risk features.

#### 2.3.1. Aspirin

Aspirin irreversibly inhibits cyclooxygenase‐1 (COX‐1) (antiplatelet) and COX‐2 (anti‐inflammatory) enzymes, thereby decreasing TXA2 synthesis, which is a potent vasoconstrictor and promoter of platelet aggregation. This irreversible inhibition lasts for the lifespan of platelets (7–10 days). Aspirin enables rapid absorption (bioavailability 30%–40%) with plasma peak levels within 30–40 min and widespread distribution throughout the body. The high on‐treatment platelet reactivity (HTPR) to aspirin is more pronounced in CKD patients, leading to impaired aspirin responsiveness [17, 18]. Gastrointestinal (GI) bleeding, ulceration, allergic reactions, and hypersensitivity are among the most frequent adverse effects of aspirin. A meta‐analysis of 22 trials involving over 75,000 patients found that low‐dose aspirin (75–325 mg/day) significantly reduced GI bleeding, with a relative risk of 2.07 (95% CI 1.61–2.66) [19]. A population‐based study of 14,627 patients found that aspirin plus a proton pump inhibitor (PPI) reduced recurrent major GI hospitalizations (0.125 vs. 0.103 per person‐year compared with aspirin alone) and was more cost‐effective for high‐risk GI patients on long‐term antiplatelet therapy [20]. Accordingly, the ACC/AHA guidelines recommend that aspirin users with a history of GI issues should also receive PPI therapy [21]. Previous randomized trials and the ISIS‐2 trial established the efficacy of aspirin in reducing vascular death and reinfarction [22, 23]. Despite its foundational role, aspirin’s limitations—including residual platelet reactivity in certain populations and the absence of a reversible mechanism—have prompted the exploration of alternative or adjunctive strategies.

#### 2.3.2. Dipyridamole and Cilostazol

Dipyridamole and cilostazol are phosphodiesterase inhibitors that work by increasing intracellular cyclic adenosine monophosphate (cAMP), leading to antiplatelet effects and vasodilation [17, 18]. Dipyridamole has not been widely used as a primary treatment for IHD due to inconclusive data regarding its efficacy in preventing coronary events. It is mainly used in ischemic cerebrovascular disease as evidenced by the ESPS2 study and ESPRIT trial [24, 25]. On the other hand, cilostazol is primarily indicated for the treatment of intermittent claudication in patients with peripheral arterial disease.

#### 2.3.3. P2Y12 Inhibitors

P2Y12 receptor antagonists (ticlopidine, clopidogrel, ticagrelor, and prasugrel) inhibit ADP‐mediated platelet activation. In addition, P2Y12 inhibitors modulate inflammation, stabilize atherosclerotic plaques, prevent vasoconstriction, decrease reperfusion injury, and have pleiotropic effects on endothelial function and hemostatic profiles [26]. Ticlopidine, the first P2Y12 inhibitor, was used in the early 1990s. However, because of the potentially serious side effects of hematological toxicity, ticlopidine was later replaced by next‐generation P2Y12 inhibitors, clopidogrel, ticagrelor, and prasugrel [27]. Cangrelor is an intravenous, reversible P2Y12 receptor antagonist that is primarily used during PCI [17, 18]. A meta‐analysis conducted by Chiarito et al. [28] in 2020 showed that P2Y12 inhibitor monotherapy is associated with a lower risk of MI in patients with established atherosclerotic cardiovascular disease (ASCVD). The PANTHER meta‐analysis, including 24,325 patients with established CAD, demonstrated that P2Y12 inhibitor monotherapy (mainly clopidogrel and ticagrelor) following DAPT provides better protection against MACEs over 2 years compared to aspirin (HR [hazard ratio] 0.88; 95% CI 0.79–0.97; *p* = 0.012). It offers comparable overall bleeding rates but fewer GI bleeding events and hemorrhagic strokes [29].

Moreover, several other meta‐analyses confirmed the noninferior or superior efficacy of P2Y12 inhibitors compared with aspirin in reducing MACEs. The 2020 MODEL U‐SES prospective single‐arm registry showed noninferiority in 1‐year outcomes (primary endpoint 4.3% vs. 5.5%; *p* < 0.0001) with P2Y12 inhibitors, while the 2016 Korean registry found that clopidogrel reduced recurrent ischemic events (HR 0.54; 2.6% vs. 3.8%; *p* = 0.02) with similar bleeding rates [30, 31]. A meta‐analysis of nine randomized trials with 61,623 patients demonstrated that P2Y12 inhibitor monotherapy (clopidogrel or ticagrelor) considerably reduced MACEs by 11% (RR 0.89; 95% CI 0.84–0.95) and MI by 19% (RR 0.81; 95% CI 0.71–0.92) [32]. A systematic review and meta‐analysis by Al‐Abdouh et al. [33] in 2022, with 56,982 patients, showed that P2Y12 inhibitors significantly reduced MI risk (RR 0.83; 95% CI 0.72–0.94) and stroke (RR 0.90; 95% CI 0.83–0.99). The 2021 Bayesian network meta‐analysis proved that early de‐escalation of DAPT (1–3 months) to P2Y12 inhibitor monotherapy remarkably reduced total bleeding events—HRs of 0.28 for 1 month and 0.57 for 3 months—without increasing ischemic risks [34]. A network meta‐analysis involving 73,126 PCI patients revealed that aspirin monotherapy after DAPT was associated with a significantly higher risk of MI compared with P2Y12 inhibitor monotherapy (RR 1.32; 95% CI 1.08–1.62). P2Y12 inhibitors also favorably trended toward preventing stent thrombosis and stroke. In these studies, there was no significant difference in stroke, all‐cause mortality, or major bleeding [35]. In 2023, a meta‐analysis of 24,460 post‐PCI patients found that P2Y12 inhibitor monotherapy significantly lowered the risk of MACEs compared to aspirin (OR 0.70; 95% CI 0.60–0.80; *p* < 0.00001), primarily driven by reductions in revascularization and stroke. The safety profile showed similar major bleeding risks between groups (OR 0.86; *p* = 0.54), though ticagrelor monotherapy was associated with a borderline increased bleeding risk (OR 1.81; *p* = 0.05) [36]. These studies conclude that P2Y12 inhibitor monotherapy is a safe and effective strategy for PCI patients. Overall, P2Y12 inhibitors may offer superior efficacy without substantially increasing bleeding risk, with ticagrelor requiring cautious use due to bleeding concerns [37]. A notable limitation across these meta‐analyses is the significant heterogeneity in patient populations, DAPT durations, and definitions of bleeding outcomes, which complicates the formulation of a unified clinical strategy.

##### 2.3.3.1. Clopidogrel

Clopidogrel, a second‐generation oral P2Y12 inhibitor, is a thienopyridine prodrug that requires two‐step hepatic CYP2C19 activation to form an active metabolite that irreversibly inhibits the platelet P2Y12 receptor. This delays its onset of action, and genetic polymorphism affecting CYP2C19 activity varies individual pharmacokinetic responses to clopidogrel. HTPR to clopidogrel represents a significant clinical challenge, affecting up to 30%–40% of patients depending on the population studied. HTPR has been associated with an increased risk of stent thrombosis and recurrent ischemic events, particularly in the early post‐PCI period. The prevalence of HTPR is even higher in specific subgroups, including patients with DM and CKD and those carrying CYP2C19 loss‐of‐function alleles. HTPR to clopidogrel has been reported to be as high as 84% in patients with advanced CKD [17, 18]. Recent evidence suggests that early identification of HTPR through platelet function testing or genetic screening may enable timely switching to more potent P2Y12 inhibitors, potentially improving outcomes. In a study examining patients undergoing coronary interventions, HTPR was identified in approximately one‐third of patients and was independently associated with an increased risk of 6‐month MACEs, highlighting the importance of recognizing and addressing this phenomenon in clinical practice. This underscores the potential value of tailored antiplatelet strategies based on individual response profiles [38]. The efficacy of clopidogrel in improving endothelial function, enhancing ADP‐mediated platelet inhibition, and reducing coagulation activity has been demonstrated in the I‐LOVE‐MONO trial, a randomized, open‐labeled, two‐period crossover study [26]. In 2004, the CADET trial proved that clopidogrel was more effective than aspirin in reducing thrombotic risk factors and C‐reactive protein (CRP) levels after AMI [39]. In ACS, the loading dose of 300–600 mg achieves rapid platelet inhibition within a 2‐h onset, with a maintenance dose of 75 mg daily. Although clopidogrel causes less gastric mucosal inflammation than aspirin, the 2007 ACC/AHA guidelines for ACS suggested concurrent use of PPIs due to impaired hemostasis in patients with a history of GI hemorrhage [40].

The CAPRIE trial, established in 1996, was the first randomized, double‐blind study that demonstrated the efficacy of clopidogrel in IHD. It involved 19,185 participants with ASCVD. In this study, long‐term clopidogrel monotherapy modestly reduced the risk of ischemic stroke, MI, or vascular death by 8.7% compared to aspirin (5.32% vs. 5.83%; *p* = 0.043) with less GI adverse effects [41]. After that, in 2001, the CURE trial studied 12,562 patients with unstable angina or non‐ST elevation myocardial infarction (NSTEMI). This trial demonstrated that adding clopidogrel to aspirin (DAPT) significantly reduced the incidence of cardiovascular death, nonfatal MI, or stroke (9.3% compared to 11.4%; *p* < 0.001). Furthermore, patients treated with clopidogrel experienced significantly lower rates of in‐hospital refractory or severe ischemia, heart failure, and revascularization procedures (16.5% vs. 18.8%; *p* < 0.001), although there was an increased risk of major bleeding (3.7% vs. 2.7%; *p* = 0.001), pointing to the need to balance efficacy and safety profiles [42]. In the 2004 MATCH trial, clopidogrel monotherapy yielded better antithrombotic activity in the elderly with reduced incidence of bleeding [43]. The 2019 KAMIR‐NIH study of 1819 DES‐treated MI patients evidenced that after 12 months of DAPT, clopidogrel monotherapy showed similar efficacy and safety compared to aspirin (0.7% each; HR 1.06; *p* = 0.923) [44]. Moreover, in 2020, the open‐label, randomized controlled POPular AGE trial, which compared clopidogrel versus ticagrelor or prasugrel in patients aged over 70 years with NSTE‐ACS, showed that clopidogrel led to fewer bleeding events without an increase in all‐cause death, MI, and stroke [45].

In 2021, the HOST‐EXAM study, conducted at 37 South Korean sites with 5438 post‐PCI patients, confirmed that 24‐month clopidogrel monotherapy significantly reduced the composite endpoint of all‐cause death, MI, stroke, ACS readmission, and Bleeding Academic Research Consortium (BARC) type ≥ 3 bleeding compared to aspirin (HR 0.68; 95% CI 0.52–0.87; *p* = 0.003). Notably, clopidogrel showed superior efficacy in patients with and without diabetes, with lower event rates (6.3% vs. 9.2% in diabetics; HR 0.69; *p* = 0.03) [46]. The HOST‐EXAM study group was followed for a median of 5.8 years (HOST‐EXAM Extended Study). It demonstrated that clopidogrel monotherapy significantly reduced the risk of the primary composite endpoint (12.8% vs. 16.9%; HR 0.74; *p* < 0.001), thrombotic events (7.9% vs. 11.9%; HR 0.66; *p* < 0.001), and major bleeding (4.5% vs. 6.1%; HR 0.74; *p* = 0.016) compared to aspirin. The findings support the sustained benefit of clopidogrel monotherapy for secondary prevention post‐PCI over extended periods [47]. Thus, clopidogrel is approved and recommended for elderly patients with aspirin intolerance, particularly in resource‐limited settings, making it an attractive alternative P2Y12 inhibitor for those at higher bleeding risk. However, the HOST‐EXAM population consisted predominantly of East Asian patients, who exhibit different pharmacogenetic profiles and bleeding risks compared to Western populations, limiting the global generalizability of these findings without additional validation in diverse cohorts.

##### 2.3.3.2. Ticagrelor

Ticagrelor, a cyclopentyl triazolopyrimidine, is the first reversible, direct‐acting platelet P2Y12‐ADP receptor inhibitor, which is metabolized by cytochrome P450 (CYP3A4/5) to form its active metabolite. It has a rapid onset of action with declining plasma concentrations at about 12 h, requiring a twice‐daily dose. Ticagrelor exhibits lower HTPR, and its antiplatelet response remains consistent during hemodialysis [17, 18]. In the ONSET/OFFSET study, ticagrelor demonstrated more rapid and greater platelet inhibition with a loading dose of 180 mg, with its antiplatelet effects sustained during the maintenance phase at 90 mg twice daily [48]. In 2009, the superior efficacy of ticagrelor in ACS was first confirmed in the PLATO trial, a multicenter, double‐blind, randomized study, which showed that ticagrelor significantly reduced the composite endpoint of cardiovascular death, MI, or stroke compared to clopidogrel, although it was associated with an increased risk of nonprocedural bleeding. Additionally, dyspnea was identified as a common side effect in the PLATO trial, although most cases were mild or transient, lasting less than 24 h [49]. Critically, the PLATO trial has been subject to controversy regarding regional variation in outcomes—particularly the unexpected finding of higher mortality with ticagrelor in the United States compared to the rest of the world, which has been attributed to potential confounding factors including aspirin dosing and differences in patient management, underscoring the importance of considering geographic and practice variations when interpreting trial results. After that, in 2015, the PEGASUS‐TIMI 54 trial evaluated long‐term ticagrelor (90 or 60 mg twice daily) plus aspirin versus placebo in 21,162 patients with prior MI. Over a median follow‐up of 33 months, ticagrelor remarkably lowered cardiovascular mortality and morbidity (7.85% vs. 9.04%; HR 0.85; *p* = 0.008), with higher major bleeding (2.60% vs. 1.06%; *p* < 0.001). The 60‐mg dose showed similar efficacy with fewer bleeding events [50].

The 2019 THEMIS trial, a double‐blind, randomized controlled study involving 19,220 patients with Type 2 diabetes and stable CAD (without prior MI or stroke), found that adding ticagrelor to aspirin reduced the composite of cardiovascular death, MI, or stroke from 8.5% to 7.7% (HR 0.90; *p* = 0.04) but increased major bleeding from 2.56% to 3.95% [51]. The THEMIS‐PCI trial focused on patients with diabetes, stable CAD, and a history of PCI, demonstrating that ticagrelor reduced ischemic events from 2.64% to 3.80% per 100 patient‐years, albeit with a higher bleeding risk, suggesting a potential net benefit in this subgroup [52]. Overall, these results underscore the importance of individualized risk assessment when considering ticagrelor therapy for patients with stable CAD and diabetes. The 2016 TWILIGHT trial was the first randomized, double‐blind, placebo‐controlled study demonstrating that after 3 months of DAPT (ticagrelor plus aspirin), switching to ticagrelor monotherapy significantly reduced bleeding risk (4.0% vs. 7.1%; HR 0.56; *p* < 0.001) without increasing the risk of ischemic events [53]. The 2018 GLOBAL LEADERS trial found that a 1‐month course of DAPT followed by ticagrelor monotherapy was noninferior to 12 months of DAPT in preventing death or Q‐wave MI over 2 years (HR 0.87; 95% CI 0.75–1.01; *p* = 0.073) [54]. Similarly, the 2020 TICO trial demonstrated that switching to ticagrelor monotherapy after 3 months of DAPT in ACS patients with DES significantly reduced net adverse clinical events (3.9% vs. 5.9%; HR 0.66; *p* = 0.01), primarily due to decreased major bleeding (1.7% vs. 3.0%; HR 0.56; *p* = 0.02), without increasing ischemic events [55]. This evidence supports the efficacy and safety of short‐term DAPT while maintaining ticagrelor monotherapy. Moreover, ticagrelor was associated with better saphenous vein graft (SVG) patency after coronary artery bypass graft (CABG) as evidenced by the DACAB trial [56].

##### 2.3.3.3. Prasugrel

Prasugrel, a thienopyridine that irreversibly inhibits the P2Y12‐ADP receptor, offers more potent and consistent platelet inhibition compared to other agents, with a rapid onset of action typically within 15–30 min with a 60‐mg loading dose and 10 mg daily (5 mg daily in elderly or low‐weight patients) [17, 18]. In the TRITON‐TIMI 38 trial, which directly compared prasugrel to clopidogrel—both in combination with aspirin—in patients with ACS undergoing scheduled PCI, prasugrel significantly reduced the incidence of ischemic events, including stent thrombosis, although it increased the risk of major fatal bleeding [57]. Conversely, a Phase 2, randomized, dose‐ranging, double‐blind safety trial—the JUMBO‐TIMI 26—found no statistically significant difference in bleeding rates between prasugrel and clopidogrel (1.7% vs. 1.2%; HR 1.42; 95% CI 0.40–5.08) [58]. Nonetheless, caution should be exercised when prescribing prasugrel, particularly in elderly patients and those with a history of stroke or transient ischemic attack (TIA), due to its heightened bleeding risk in these populations. The exclusion of elderly patients and those with low body weight from the TRITON‐TIMI 38 trial’s primary analysis represents a significant limitation, as these populations constitute a substantial proportion of real‐world patients with ACS, and subsequent analyses have suggested that dose reduction may mitigate bleeding risk without compromising efficacy. Table 1 compares the pharmacological profiles of various oral antiplatelet agents.

**Table 1:** Pharmacological profiles of oral antiplatelets.

	Aspirin	Clopidogrel	Ticagrelor	Prasugrel
Drug class	NSAID	Thienopyridine (prodrug)	Cyclopentyl triazolopyrimidine	Thienopyridine (prodrug)
Site of action	Irreversible COX‐1 and COX‐2 inhibition	Irreversible P2Y12 inhibition	Reversible P2Y12 inhibition	Irreversible P2Y12 inhibition
Cytochrome P450 involvement	Minimal; mainly irrelevant	CYP2C19	CYP3A4/5	CYP2C19
Metabolic activation	No	Yes	No	Yes
Onset of action	Rapid	2–8 h (> 6 h with a 300‐mg loading dose, 2–4 h with a 600‐mg loading dose)	Rapid (30 min–4 h)	Rapid (30 min–4 h; within 30 min with a 60‐mg loading dose)
Typical dose	300‐mg loading dose; 75 mg daily	300–600‐mg loading dose; 75 mg daily	180‐mg loading dose; 90 mg twice daily	60‐mg loading dose; 10 mg daily
Dosing frequency	Once daily	Once daily	Twice daily	Once daily
High on‐treatment platelet reactivity (HTPR)	More common in CKD patients	Higher HTPR, especially in CYP2C19 poor metabolizers and CKD patients	Lower HTPR and more consistent response	Lower HTPR compared to clopidogrel
Platelet response during hemodialysis (HD)	N/A	Reduced HTPR, response varies	Unchanged response during HD	Limited data, generally stable
Common side effects	Bleeding, GI upset	Bleeding	Bleeding, dyspnea	Bleeding, fewer GI issues

### 2.4. Glycoprotein IIb/IIIa Inhibitors

Glycoprotein IIb/IIIa inhibitors (abciximab, eptifibatide, and tirofiban) are intravenous antiplatelet agents that are typically used in acute settings such as during PCI or ACS [58]. Table 2 summarizes the pharmacotherapeutic profiles of various intravenous antiplatelets.

**Table 2:** Pharmacotherapeutic profiles of various intravenous antiplatelets.

	Cangrelor	Abciximab	Eptifibatide	Tirofiban
Drug class	Adenosine triphosphate analog	Monoclonal antibody	Cyclic heptapeptide	Small, nonpeptide molecule
Site of action	Reversible P2Y12 inhibition	Glycoprotein IIb/IIIa receptor inhibitor	Glycoprotein IIb/IIIa receptor inhibitor	Glycoprotein IIb/IIIa receptor inhibitor
Typical dose	Cangrelor dose: Bolus 30 mcg/kg (0.03 mg/kg) injection, then 4 mcg/kg/min (0.004 mg/kg/min) infusion	Bolus 0.25 mg/kg injection, then 0.125 *μ*g/kg/min infusion	Bolus 180 *μ*g/kg (10 min) + 180 *μ*g/kg Infusion: 2 *μ*g/kg/min	Bolus 25 *μ*g/kg (over 3 min) Infusion: 0.15 *μ*g/kg/min
Renal adjustment	No	No	Yes	Yes

### 2.5. Recommendations and Guidelines

DAPT with aspirin and a P2Y12 inhibitor is the mainstay for secondary prevention of ischemic events, especially after PCI. In patients with stable CAD who undergo PCI, the AHA and ESC guidelines recommend a minimum of 1 month of DAPT after bare metal stent (BMS) implantation (Class IA) and 6 months after DES (Class IB), with a reduction to 3 months for those at HBR. For patients with ACS, at least 12 months of DAPT is advised following PCI with DES [59, 60]. Additionally, the 2019 ESC guideline for managing chronic coronary syndrome (CCS) suggests adding vascular‐dose rivaroxaban (2.5 mg twice daily) or a P2Y12 inhibitor to aspirin for patients at moderate to high ischemic risk, a strategy supported by evidence from the COMPASS trial [37, 61]. According to the DAPT study, prolonged DAPT (aspirin plus either clopidogrel or prasugrel) beyond 12 months significantly reduced stent thrombosis (0.4% vs. 1.4%; HR 0.29; *p* < 0.001) and MACEs (4.3% vs. 5.9%; HR 0.71; *p* < 0.001), although it increased moderate or severe bleeding (2.5% vs. 1.6%; HR 1.61; *p* = 0.001). All‐cause mortality was marginally higher with extended therapy (2.0% vs. 1.5%; HR 1.36; *p* = 0.05) [62]. Again, the SMART‐CHOICE trial in 2019 showed that 3‐month DAPT was noninferior to prolonged DAPT with a significantly lower bleeding risk in ACS patients [63]. The STOPDAPT‐1 and STOPDAPT‐2 trials collectively support the safety and efficacy of shortened DAPT durations following PCI with DES. The STOPDAPT‐1 trial demonstrated that 3‐month DAPT followed by aspirin monotherapy was as safe as the conventional 12‐month DAPT, with similar clinical outcomes. Building on this, the STOPDAPT‐2 trial showed that 1‐month DAPT followed by clopidogrel monotherapy was noninferior to 12‐month DAPT, with a significant reduction in major bleeding (HR 0.26; *p* = 0.004) and a trend toward fewer cardiovascular events (HR 0.77; *p* = 0.03). Together, these studies suggest that abbreviated DAPT regimens—particularly 1–3 months—are effective and potentially safer options in the era of new‐generation DES [64, 65]. Having said that, the optimal duration of DAPT, particularly after complex PCI, should be determined based on ischemic and bleeding risks utilizing risk stratification tools such as the PRECISE‐DAPT score [66]. The PRECISE‐DAPT score has emerged as a validated and practical tool for guiding DAPT duration decisions. This risk score integrates five clinical variables (age, hemoglobin, white blood cell count, creatinine clearance, and prior spontaneous bleeding) to predict the risk of out‐of‐hospital bleeding during DAPT. Its predictive value extends beyond bleeding outcomes; recent evidence demonstrates that the PRECISE‐DAPT score also predicts in‐hospital mortality in patients with STEMI undergoing primary PCI, with higher scores correlating with increased mortality risk [67]. The score’s utility lies in its ability to identify patients who would derive net benefit from abbreviated DAPT (score ≥ 25) versus those who may warrant extended therapy (score < 25). Clinical adoption of this score has been endorsed by both the ESC and AHA/ACC guidelines, representing a shift toward more objective, evidence‐based DAPT duration selection. After the recommended duration of DAPT, the transition to single antiplatelet therapy with either aspirin or a P2Y12 inhibitor should be individualized. A meta‐analysis by Liu et al. [68] demonstrated that early discontinuation of aspirin and continuation of P2Y12 inhibitor monotherapy can effectively reduce ischemic events.

### 2.6. Antiplatelet Therapy in Special Populations

Managing antiplatelet therapy in groups such as patients with CKD, the elderly, those with diabetes, and individuals with atrial fibrillation requires careful assessment of individual risks for both thrombosis and bleeding. Personalized strategies are essential to optimize cardiovascular protection while minimizing potential adverse effects.

#### 2.6.1. Antiplatelet Therapy in Patients With CKD

CKD is associated with a higher risk of thrombosis due to the accumulation of uremic toxins, persistent low‐grade inflammation, impaired vascular integrity, enhanced platelet activity, and a hypercoagulable state [69]. At the same time, altered platelet function and adhesion disturbances in CKD increase the risk of both spontaneous bleeding and bleeding induced by antiplatelet therapy [70]. In patients with CKD, antiplatelet treatment has been shown to effectively reduce MACEs and dialysis vascular access failures [71]. However, the efficacy of aspirin in CKD patients is often suboptimal because of high residual platelet reactivity. Additionally, as an NSAID, aspirin carries potential nephrotoxicity; even at a low dose of 75 mg daily, it can cause a temporary decline in renal function, which typically recovers after stopping the medication [72].

Furthermore, the pharmacokinetics of clopidogrel are significantly affected by CKD, leading to increased HTPR. In contrast, the relative efficacy and safety of prasugrel versus ticagrelor do not appear to be influenced by estimated glomerular filtration rate (eGFR) [70]. A recent systematic review and meta‐analysis demonstrated that in ACS patients with CKD, ticagrelor‐based DAPT is superior to clopidogrel‐based DAPT, showing a reduced rate of MACEs (RR 0.89; 95% CI 0.80–0.99; *p* = 0.04) without significant differences in major bleeding or all‐cause mortality [69]. The TWILIGHT‐CKD subgroup analysis also revealed that ticagrelor monotherapy reduces bleeding risk compared to DAPT without compromising ischemic protection [53]. Therefore, antiplatelet therapy in CKD should be tailored to each patient, carefully balancing the reduction of ischemic events against the risk of bleeding.

#### 2.6.2. Antiplatelet Therapy in Elderly Patients

Elderly patients are at increased risk for both thrombotic and bleeding events due to an imbalance between coagulation and fibrinolysis, increased blood stasis, oxidative stress, and age‐related collagen and amyloid deposits in the arterial wall that contribute to endothelial dysfunction. Additionally, increased platelet reactivity predisposes patients to thrombosis. Factors such as poor medication adherence, age‐related changes in organ function, and polypharmacy‐related drug interactions can also influence the pharmacological response to antiplatelet agents, making individualized treatment essential [73].

Aspirin remains the foundation of secondary prevention, with a meta‐analysis showing that aspirin provided a 10% absolute risk reduction in vascular events over 5 years in patients aged 65–74, with a minimal increase in bleeding risk (0.5%) [22]. Among P2Y12 inhibitors, clopidogrel is widely recommended for secondary prevention in the elderly due to its favorable safety profile. The POPular AGE trial proved the efficacy of clopidogrel in decreasing bleeding events (17.6% vs. 23.1%; OR 0.74; 95% CI 0.56–0.97) without increasing thrombotic events (12.8% vs. 12.5%; OR 1.02; 95% CI 0.72–1.45) compared to ticagrelor in patients aged ≥ 75 years [45]. However, its efficacy may be lowered in the elderly due to the high prevalence of HTPR. Potent P2Y12 inhibitors like ticagrelor and clopidogrel show superior efficacy in ischemic event prevention but with bleeding risks. In the PLATO trial, ticagrelor reduced cardiovascular mortality and ischemic endpoints in patients over 75 years but at the expense of increased bleeding [49]. The SWEDEHEART registry studied patients aged 80 years who were on aspirin‐based DAPT with either clopidogrel or ticagrelor post‐MI. In this registry, ticagrelor had notably higher mortality and bleeding than clopidogrel [74].

In addition, standard‐dose prasugrel (10 mg) is contraindicated in patients aged 75 and older due to a 32% increase in major bleeding observed in the TRITON‐TIMI 38 trial [57]. However, a lower dose of 5 mg/day may offer more effective platelet inhibition than clopidogrel without significantly increasing bleeding risk. The comparable efficacy of low‐dose prasugrel versus clopidogrel has been demonstrated in the TRILOGY ACS study (medically managed elderly ACS patients) and ELDERLYACS 2 trial (invasively managed ACS elderly patients) [75, 76]. Similarly, in the subgroup analysis of the ISAR‐REACT 5 trial, a reduced dose of prasugrel was associated with similar anti‐ischemic benefit without increasing bleeding risk in the elderly (over 75 years) or those with low body weight (under 60 kg) [77]. For elderly patients with isolated HBR, 1‐month DAPT is noninferior to standard DAPT in preventing ischemic events and significantly reducing bleeding (6.5% vs. 9.4%; 95% CI −4.40 to −1.24; *p* < 0.001 for superiority), as evidenced in the MASTER DAPT trial, with clopidogrel preferred over aspirin after DAPT cessation [78]. In cases of HBR combined with high thrombotic risk, using low‐dose prasugrel plus aspirin for the first 2–3 months after ACS, then transitioning to clopidogrel, is a reasonable option because thrombotic risk is highest initially and gradually decreases over time, while bleeding risk remains constant [79].

#### 2.6.3. Antiplatelet Therapy in Patients With DM

Patients with DM exhibit heightened platelet reactivity due to dysregulated signaling pathways, leading to increased platelet activation and aggregation. Moreover, patients with diabetes have more extensive coronary heart disease and advanced Killip class heart failure, leading to high cardiovascular mortality compared to those without diabetes (5.5 vs. 3.3%; HR 1.7 [0.99–2.8]; *p* = 0.054) [76]. As a result, third‐generation P2Y12 inhibitors such as prasugrel and ticagrelor are particularly beneficial in this population. Notably, in the TRITON‐TIMI 38 trial, prasugrel demonstrated a greater net clinical benefit over clopidogrel among diabetic patients, with event rates of 14.6% versus 19.2% (HR 0.74; *p* = 0.001) [56]. However, long‐term DAPT does not significantly reduce MACEs in DM patients after PCI and is associated with an increased risk of bleeding [80].

The complexity of antiplatelet management in diabetic patients is further amplified in specific interventional contexts, such as following small‐diameter stent implantation (≤ 2.5 mm). These stents are associated with higher rates of restenosis and stent thrombosis due to lower shear stress patterns and reduced luminal dimensions, creating a prothrombotic environment. In diabetic patients, the combination of impaired endothelial function, heightened platelet reactivity, and the mechanical challenges of small stents creates a uniquely high‐risk scenario. Evidence suggests that these patients may benefit from more potent P2Y12 inhibition and careful consideration of DAPT duration, as the balance between ischemic and bleeding risks is particularly delicate [81]. This represents a specific clinical niche where personalized antiplatelet strategies are paramount, yet prospective data remain limited.

#### 2.6.4. Antiplatelet Therapy in Patients With Atrial Fibrillation

In patients requiring oral anticoagulation for another indication (e.g., atrial fibrillation), a short course of triple therapy with aspirin, clopidogrel, and a direct oral anticoagulant (DOAC) for up to 1 month post‐PCI is recommended to reduce early stent thrombosis, as advised by the ESC (Class IIa, Level B) and AHA/ACC (Class I) guidelines [60]. For patients at HBR, aspirin should be discontinued after 1 week. After a brief period of triple therapy, patients should receive DOAC plus clopidogrel for 6–12 months, followed by DOAC monotherapy, with the duration of clopidogrel tailored according to bleeding risk [82].

### 2.7. Future Research Direction

Recent advances in antiplatelet therapy focus on novel agents that precisely target various steps of platelet activation, aggregation, and adhesion. These include reversible, direct‐acting P2Y12 inhibitors like elinogrel; protease‐activated receptor‐1 (PAR‐1) antagonists such as vorapaxar (intravenous) and atopaxar (oral); PAR‐4 antagonists like BMS‐986120 and BMS‐986141; collagen receptor inhibitors such as revacept (intravenous); collagen and ristocetin inhibitors like DZ‐697b; GPIb‐V‐IX complex inhibitors like anfibatide; 12‐lipoxygenase inhibitors such as VLX‐1005; TXA2 receptor antagonists like terutroban; and von Willebrand factor inhibitors including ARC1779, ALX‐0081, and GPG‐290. These agents are currently under ongoing clinical trials, and their long‐term benefits and safety profiles in IHD require validation through larger, randomized studies [27].

Beyond novel agents, the future of antiplatelet therapy lies in precision medicine approaches that integrate biomarker and genetic information into clinical decision‐making. Genotype‐guided antiplatelet therapy, particularly targeting CYP2C19 loss‐of‐function alleles that impair clopidogrel activation, represents a promising frontier. While prospective trials such as TAILOR‐PCI have shown mixed results regarding the superiority of genotype‐guided selection over standard therapy, emerging data suggest that certain subgroups—particularly those at highest ischemic risk—may derive meaningful benefit from this approach. The development of rapid point‐of‐care genetic testing platforms may facilitate broader adoption of this strategy in acute settings.

Similarly, biomarker‐guided strategies beyond genetics, including platelet function testing to identify HTPR and inflammatory biomarkers such as high‐sensitivity CRP to guide anti‐inflammatory therapy selection, are gaining traction. The integration of these approaches into a comprehensive risk stratification framework—combining clinical risk scores (e.g., PRECISE‐DAPT), genetic information, and functional testing—may enable truly personalized antiplatelet regimens that maximize efficacy while minimizing harm. Large‐scale randomized controlled trials evaluating these integrated strategies are needed before widespread clinical implementation can be recommended.

Additionally, the intersection of antiplatelet therapy with emerging cardiovascular treatments, including novel lipid‐lowering agents (PCSK9 inhibitors, inclisiran, and Lp(a)‐targeted therapies) and anti‐inflammatory drugs (colchicine), warrants investigation. Understanding the potential synergistic or additive effects of these combination strategies on platelet function, thrombosis risk, and clinical outcomes will be essential as polypharmacy becomes increasingly common in secondary prevention. The optimal sequencing and de‐escalation of these multiple preventive therapies also require systematic study.

## 3. Conclusion

In conclusion, antiplatelet therapy is integral in preventing recurrent ischemic events and enhancing overall prognosis following ACS. Following PCI with DES, DAPT, typically comprising aspirin and a P2Y12 inhibitor, has demonstrated proven effectiveness in reducing stent thrombosis and MACEs. Advances in pharmacotherapy have enabled a shorter duration of DAPT, with a transition to single antiplatelet therapy—particularly with P2Y12 inhibitors such as ticagrelor or clopidogrel—showing superior efficacy and a favorable bleeding profile compared to aspirin alone. The duration of DAPT, choice of therapy, and optimal timing for transition to monotherapy should be personalized depending on the individual’s ischemic and bleeding risks. Validated risk stratification tools such as the PRECISE‐DAPT score facilitate objective decision‐making, while recognition of HTPR—particularly in high‐risk subgroups such as diabetics, patients with CKD, and those undergoing small‐diameter stent implantation—enables identification of individuals who may benefit from intensified or alternative antiplatelet strategies. The future of antiplatelet therapy lies in precision medicine approaches integrating genetic, functional, and clinical biomarkers to achieve truly individualized treatment. As the field continues to evolve, ongoing research will refine these strategies, ultimately improving outcomes for the millions of patients worldwide living with established CVD.

## Author Contributions

**May Thu Kyaw:** conceptualization, data curation, formal analysis, investigation, methodology, project administration, resources, supervision, validation, visualization, writing – original draft preparation, writing – review and editing. **Arkar Moe:** conceptualization, resources, supervision, validation.

## Funding

No funding was received for this manuscript.

## Ethics Statement

The authors have nothing to report.

## Consent

The authors have nothing to report.

## Conflicts of Interest

The authors declare no conflicts of interest.

## Data Availability

Data sharing is not applicable to this article as no new data were created or analyzed in this study.

## References

[1] Khan M. A. , Hashim M. J. , Mustafa H. , Baniyas M. Y. , Al Suwaidi S. K. B. M. , AlKatheeri R. , Alblooshi F. M. K. , Almatrooshi M. E. A. H. , Alzaabi M. E. H. , Al Darmaki R. S. , and Lootah S. N. A. H. , Global Epidemiology of Ischemic Heart Disease: Results From the Global Burden of Disease Study, Cureus. (2020) 12, no. 7, 10.7759/cureus.9349, 32742886. PMC7384703 32742886

[2] Vazquez-Oliva G. , Zamora A. , Ramos R. , Marti R. , Subirana I. , Grau M. , Degano I. R. , Marrugat J. , and Elosua R. , Acute Myocardial Infarction Population Incidence and Mortality Rates, and 28-Day Case-Fatality in Older Adults. The REGICOR Study, Revista Española de Cardiología. (2018) 71, no. 9, 718–725, 10.1016/j.rec.2017.10.019, 29174866. 29174866

[3] Law M. R. , Watt H. C. , and Wald N. J. , The Underlying Risk of Death After Myocardial Infarction in the Absence of Treatment, Archives of Internal Medicine. (2002) 162, no. 21, 2405–2410, 10.1001/archinte.162.21.2405, 12437397. 12437397

[4] Bishu K. G. , Lekoubou A. , Kirkland E. , Schumann S. O. , Schreiner A. , Heincelman M. , Moran W. P. , and Mauldin P. D. , Estimating the Economic Burden of Acute Myocardial Infarction in the US: 12 Year National Data, American Journal of the Medical Sciences. (2020) 359, no. 5, 257–265, 10.1016/j.amjms.2020.02.004, 32265010. 32265010

[5] Vargas I. , Wickline S. A. , and Pan H. , The Role of Thrombosis and Vessel Injury in Acute Myocardial Infarction: Current Standard of Care and Therapeutic Options, Cardiology and Cardiovascular Medicine. (2021) 5, no. 5, 502–529, 10.26502/fccm.92920217.

[6] Palasubramaniam J. , Wang X. , and Peter K. , Myocardial Infarction-From Atherosclerosis to Thrombosis, Arteriosclerosis, Thrombosis, & Vascular Biology. (2019) 39, no. 8, e176–e185, 10.1161/ATVBAHA.119.312578, 31339782. 31339782

[7] Tatsumi K. and Mackman N. , Tissue Factor and Atherothrombosis, Journal of Atherosclerosis and Thrombosis. (2015) 22, no. 6, 543–549, 10.5551/jat.30940, 26016513. 26016513

[8] Steen D. L. , Khan I. , Andrade K. , Koumas A. , and Giugliano R. P. , Event Rates and Risk Factors for Recurrent Cardiovascular Events and Mortality in a Contemporary Post Acute Coronary Syndrome Population Representing 239 234 Patients During 2005 to 2018 in the United States, Journal of the American Heart Association. (2022) 11, no. 9, e022198, 10.1161/JAHA.121.022198, 35475346. 35475346 PMC9238606

[9] Fox K. A. , Carruthers K. , Steg P. G. , Avezum A. , Granger C. B. , Montalescot G. , Goodman S. G. , Gore J. M. , Quill A. L. , Eagle K. A. , and GRACE Investigators , Has the Frequency of Bleeding Changed Over Time for Patients Presenting With an Acute Coronary Syndrome? The Global Registry of Acute Coronary Events, European Heart Journal. (2010) 31, no. 6, 667–675, 10.1093/eurheartj/ehp499, 20007159. 20007159 PMC2838680

[10] Nair R. , Johnson M. , Kravitz K. , Huded C. , Rajeswaran J. , Anabila M. , Blackstone E. , Menon V. , Lincoff A. M. , Kapadia S. , and Khot U. N. , Characteristics and Outcomes of Early Recurrent Myocardial Infarction After Acute Myocardial Infarction, Journal of the American Heart Association. (2021) 10, no. 16, e019270, 10.1161/JAHA.120.019270, 34333986. 34333986 PMC8475017

[11] Thune J. J. , Signorovitch J. E. , Kober L. , McMurray J. J. , Swedberg K. , Rouleau J. , Maggioni A. , Velazquez E. , Califf R. , Pfeffer M. A. , and Solomon S. D. , Predictors and Prognostic Impact of Recurrent Myocardial Infarction in Patients With Left Ventricular Dysfunction, Heart Failure, or Both Following a First Myocardial Infarction, European Journal of Heart Failure. (2011) 13, no. 2, 148–453, 10.1093/eurjhf/hfq194, 21037250. 21037250

[12] Abtan J. , Bhatt D. L. , Elbez Y. , Sorbets E. , Eagle K. , Ikeda Y. , Wu D. , Hanson M. E. , Hannachi H. , Singhal P. K. , Steg P. G. , and REACH Registry Investigators , Residual Ischemic Risk and Its Determinants in Patients With Previous Myocardial Infarction and Without Prior Stroke or TIA: Insights From the REACH Registry, Clinical Cardiology. (2016) 39, no. 11, 670–677, 10.1002/clc.22583, 27588731. 27588731 PMC6490735

[13] Généreux P. , Rutledge D. R. , Palmerini T. , Caixeta A. , Kedhi E. , Hermiller J. B. , Wang J. , Krucoff M. W. , Jones-McMeans J. , Sudhir K. , and Simonton C. A. , Stent Thrombosis and Dual Antiplatelet Therapy Interruption With Everolimus-Eluting Stents: Insights From the Xience V Coronary Stent System Trials, Circulation: Cardiovascular Interventions. (2015) 8, no. 5, e001362, 10.1161/CIRCINTERVENTIONS.114.001362, 25940520. 25940520

[14] Chau K. H. , Kirtane A. J. , Easterwood R. M. , Redfors B. , Zhang Z. , Witzenbichler B. , Weisz G. , Stuckey T. D. , Brodie B. R. , Rinaldi M. J. , Neumann F. J. , Metzger D. C. , Henry T. D. , Cox D. A. , Duffy P. L. , Mazzaferri E. L. , Mehran R. , and Stone G. W. , Stent Thrombosis Risk Over Time on the Basis of Clinical Presentation and Platelet Reactivity: Analysis From ADAPT-DES, JACC Cardiovascular Interventions. (2021) 14, no. 4, 417–427, 10.1016/j.jcin.2020.12.005, 33516690. 33516690

[15] Kim B. K. , Hong M. K. , Shin D. H. , Nam C. M. , Kim J. S. , Ko Y. G. , Choi D. , Kang T. S. , Park B. E. , Kang W. C. , Lee S. H. , and RESET Investigators , A New Strategy for Discontinuation of Dual Antiplatelet Therapy: The RESET Trial (REal Safety and Efficacy of 3-Month Dual Antiplatelet Therapy Following Endeavor Zotarolimus-Eluting Stent Implantation), Journal of the American College of Cardiology. (2012) 60, no. 15, 1340–1348, 10.1016/j.jacc.2012.06.043, 22999717. 22999717

[16] Feres F. , Costa R. A. , Abizaid A. , Leon M. B. , Marin-Neto J. A. , Botelho R. V. , King S. B. , Negoita M. , Liu M. , de Paula J. E. , and Mangione J. A. , OPTIMIZE Trial Investigators. Three vs Twelve Months of Dual Antiplatelet Therapy After Zotarolimus-Eluting Stents: The OPTIMIZE Randomized Trial, Jama. (2013) 310, no. 23, 2510–2522, 10.1001/jama.2013.282183, 24177257. 24177257

[17] Varian F. L. , Parker W. A. E. , Fotheringham J. , and Storey R. F. , Treatment Inequity in Antiplatelet Therapy for Ischaemic Heart Disease in Patients With Advanced Chronic Kidney Disease: Releasing the Evidence Vacuum, Platelets. (2023) 34, no. 1, 2154330, 10.1080/09537104.2022.2154330. 36524601

[18] Thachil J. , Antiplatelet Therapy - A Summary for the General Physicians, Clinical Medicine. (2016) 16, no. 2, 152–160, 10.7861/clinmedicine.16-2-152, 27037385. 27037385 PMC4952969

[19] McQuaid K. R. and Laine L. , Systematic Review and Meta-Analysis of Adverse Events of Low-Dose Aspirin and Clopidogrel in Randomized Controlled Trials, American Journal of Medicine. (2006) 119, no. 8, 624–638, 10.1016/j.amjmed.2005.10.039, 16887404. 16887404

[20] Hsiao F. Y. , Tsai Y. W. , Huang W. F. , Wen Y. W. , Chen P. F. , Chang P. Y. , and Kuo K. N. , A Comparison of Aspirin and Clopidogrel With or Without Proton Pump Inhibitors for the Secondary Prevention of Cardiovascular Events in Patients at High Risk for Gastrointestinal Bleeding, Clinical Therapeutics. (2009) 31, no. 9, 2038–2047, 10.1016/j.clinthera.2009.09.005, 19843493. 19843493

[21] Fraker T. D. , Fihn S. D. , and 2002 Chronic Stable Angina Writing Committee; American College of Cardiology; American Heart Association , 2007 Chronic Angina Focused Update of the ACC/AHA 2002 Guidelines for the Management of Patients With Chronic Stable Angina: A Report of the American College of Cardiology/American Heart Association Task Force on Practice Guidelines Writing Group to Develop the Focused Update of the 2002 Guidelines for the Management of Patients With Chronic Stable Angina, Journal of the American College of Cardiology. (2007) 50, no. 23, 2264–2274, 10.1016/j.jacc.2007.08.002, 18061078. 18061078

[22] Antithrombotic Trialists′ (ATT) Collaboration , Baigent C. , Blackwell L. , Collins R. , Emberson J. , Godwin J. , Peto R. , Buring J. , Hennekens C. , Kearney P. , Meade T. , and Patrono C. , Aspirin in the Primary and Secondary Prevention of Vascular Disease: Collaborative Meta-Analysis of Individual Participant Data From Randomised Trials, Lancet. (2009) 373, no. 9678, 1849–1860, 10.1016/S0140-6736(09)60503-1, 19482214. 19482214 PMC2715005

[23] Second International Study of Infarct Survival , Randomised Trial of Intravenous Streptokinase, Oral Aspirin, Both, or Neither Among 17, 187 Cases of Suspected Acute Myocardial Infarction: ISIS-2. ISIS-2 (Second International Study of Infarct Survival) Collaborative Group, Lancet. (1988) 2, no. 8607, 349–360, 2899772. 2899772

[24] Diener H. C. , Cunha L. , Forbes C. , Sivenius J. , Smets P. , and Lowenthal A. , European Stroke Prevention Study 2. Dipyridamole and Acetylsalicylic Acid in the Secondary Prevention of Stroke, Journal of the Neurological Sciences. (1996) 143, no. 1-2, 1–13, 10.1016/S0022-510X(96)00308-5, 8981292. 8981292

[25] Lutsep H. L. , New Developments in Secondary Stroke Prevention: Impact of the European/Australasian Stroke Prevention in Reversible Ischemia Trial (ESPRIT) on Clinical Management, Journal of Stroke and Cerebrovascular Diseases. (2007) 16, no. 6, 263–267, 10.1016/j.jstrokecerebrovasdis.2007.07.005, 18035244. 18035244

[26] Park H. W. , Kang M. G. , Ahn J. H. , Bae J. S. , Tantry U. S. , Gurbel P. A. , and Jeong Y. H. , Effects of Monotherapy With Clopidogrel vs. Aspirin on Vascular Function and Hemostatic Measurements in Patients With Coronary Artery Disease: The Prospective, Crossover I-LOVE-MONO Trial, Journal of Clinical Medicine. (2021) 10, no. 12, 10.3390/jcm10122720, 34202960. PMC8235752 34202960

[27] Ahluwalia K. and Bhanwra S. , Antiplatelet Therapy: Present Status and Its Future Directions, International Journal of Basic and Clinical Pharmacology. (2014) 3, no. 2, 260–268, 10.5455/2319-2003.ijbcp20140402.

[28] Chiarito M. , Sanz-Sánchez J. , Cannata F. , Cao D. , Sturla M. , Panico C. , Godino C. , Regazzoli D. , Reimers B. , De Caterina R. , and Condorelli G. , Monotherapy with a P2Y_12_ Inhibitor or Aspirin for Secondary Prevention in Patients With Established Atherosclerosis: A Systematic Review and Meta-Analysis, Lancet. (2020) 395, no. 10235, 1487–1495, 10.1016/S0140-6736(20)30315-9, 32386592. 32386592

[29] Gragnano F. , Cao D. , Pirondini L. , Pirondini L. , Franzone A. , Kim H. S. , von Scheidt M. , Pettersen A. Å. R. , Zhao Q. , Woodward M. , Chiarito M. , McFadden E. P. , Park K. W. , Kastrati A. , Seljeflot I. , Zhu Y. , Windecker S. , Kang J. , Schunkert H. , Arnesen H. , Bhatt D. L. , Steg P. G. , Calabrò P. , Pocock S. , Mehran R. , Valgimigli M. , and PANTHER Collaboration , P2Y_12_ Inhibitor or Aspirin Monotherapy for Secondary Prevention of Coronary Events, Journal of the American College of Cardiology. (2023) 82, no. 2, 89–105, 10.1016/j.jacc.2023.04.051, 37407118. 37407118

[30] Kozuma K. , Kinoshita Y. , Hioki H. , Nanasato M. , Ito Y. , Yamaguchi J. , Shiode N. , Hibi K. , Tanabe K. , Ako J. , Morino Y. , and MODEL U-SES Study Investigators , 1-Year Safety of 3-Month Dual Antiplatelet Therapy Followed by Aspirin or P2Y_12_ Receptor Inhibitor Monotherapy Using a Bioabsorbable Polymer Sirolimus-Eluting Stent, Circulation Journal. (2020) 85, no. 1, 19–26, 10.1253/circj.CJ-20-0644, 33191392. 33191392

[31] Park T. K. , Song Y. B. , Ahn J. , Carriere K. C. , Hahn J. Y. , Yang J. H. , Choi S. H. , Choi J. H. , Lee S. H. , and Gwon H. C. , Clopidogrel Versus Aspirin as an Antiplatelet Monotherapy After 12-Month Dual-Antiplatelet Therapy in the Era of Drug-Eluting Stents, Circulation: Cardiovascular Interventions. (2016) 9, no. 1, e002816, 10.1161/CIRCINTERVENTIONS.115.002816, 26755571. 26755571

[32] Aggarwal D. , Bhatia K. , Chunawala Z. S. , Furtado R. H. M. , Mukherjee D. , Dixon S. R. , Jain V. , Arora S. , Zelniker T. A. , Navarese E. P. , Mishkel G. J. , Lee C. J. , Banerjee S. , Bangalore S. , Levisay J. P. , Bhatt D. L. , Ricciardi M. J. , and Qamar A. , P2Y_12_ Inhibitor Versus Aspirin Monotherapy for Secondary Prevention of Cardiovascular Events: Meta-Analysis of Randomized Trials, European Heart Journal Open. (2022) 2, no. 2, oeac019, 10.1093/ehjopen/oeac019, 35919116. 35919116 PMC9242055

[33] Al-Abdouh A. , Abusnina W. , Mhanna M. , Radideh Q. , Alzu′bi H. , Rmilah A. A. , Jabri A. , Barbarawi M. , Obeidat K. , Alabduh T. , and Michos E. D. , P2Y12 Inhibitors Versus Aspirin Monotherapy for Long-term Secondary Prevention of Atherosclerotic Cardiovascular Disease Events: A Systematic Review and Meta-Analysis, Current Problems in Cardiology. (2022) 47, no. 10, 101292, 10.1016/j.cpcardiol.2022.101292, 35764143. 35764143

[34] Khan S. U. , Khan M. Z. , Khan M. S. , Mahmood A. , Kalra A. , Kaluski E. , Michos E. D. , and Alkhouli M. , De-Escalation of Antiplatelets After Percutaneous Coronary Intervention: A Bayesian Network Meta-Analysis of Various De-Escalation Strategies, European Heart Journal-Cardiovascular Pharmacotherapy. (2021) 7, no. 3, 209–215, 10.1093/ehjcvp/pvaa025, 32271872. 32271872 PMC8599873

[35] Andò G. , De Santis G. A. , Greco A. , Pistelli L. , Francaviglia B. , Capodanno D. , De Caterina R. , and Capranzano P. , P2Y_12_ Inhibitor or Aspirin Following Dual Antiplatelet Therapy After Percutaneous Coronary Intervention, JACC Cardiovascular Interventions. (2022) 15, no. 22, 2239–2249, 10.1016/j.jcin.2022.08.009, 36423966. 36423966

[36] Gao T. , Meng C. , Wang Y. , Li S. , Bi L. , Geng Y. , and Zhang P. , P2Y_12_ Inhibitor vs Aspirin Monotherapy Following Dual Antiplatelet Therapy After Percutaneous Coronary Intervention: An Updated Meta-Analysis, Reviews in Cardiovascular Medicine. (2023) 24, no. 10, 10.31083/j.rcm2410284, 39077578. PMC11273139 39077578

[37] Knuuti J. , Wijns W. , Saraste A. , Knuuti J. , Wijns W. , Saraste A. , Capodanno D. , Barbato E. , Funck-Brentano C. , Prescott E. , Storey R. F. , Deaton C. , Cuisset T. , Agewall S. , and ESC Scientific Document Group , 2019 ESC Guidelines for the Diagnosis and Management of Chronic Coronary Syndromes, European Heart Journal. (2020) 41, no. 3, 407–477, 10.1093/eurheartj/ehz425, 31504439. 31504439

[38] Tekkeşin A. İ. , Kaya A. , Çakıllı Y. , Türkkan C. , Hayıroğlu M. İ. , Borklu E. B. , Kalenderoğlu K. , Gümüşdağ A. , Yıldırımtürk Ö. , Bozbeyoğlu E. , and Tatlısu M. A. , The First Six-Month Clinical Outcomes and Risk Factors Associated With High On-Treatment Platelet Reactivity of Clopidogrel in Patients Undergoing Coronary Interventions, Anatolian Journal of Cardiology. (2016) 16, no. 12, 967–973, 10.14744/AnatolJCardiol.2016.6855. 27271476 PMC5324919

[39] Woodward M. , Lowe G. D. , Francis L. M. , Rumley A. , Cobbe S. M. , and CADET Study Investigators , A Randomized Comparison of the Effects of Aspirin and Clopidogrel on Thrombotic Risk Factors and C-Reactive Protein Following Myocardial Infarction: The CADET Trial, Journal of Thrombosis and Haemostasis. (2004) 2, no. 11, 1934–1940, 10.1111/j.1538-7836.2004.01017.x, 15550024. 15550024

[40] Anderson J. L. , Adams C. D. , Antman E. M. , Bridges C. R. , Califf R. M. , Casey D. E. , Chavey W. E. , Fesmire F. M. , Hochman J. S. , Levin T. N. , Lincoff A. M. , American College of Cardiology , American Heart Association Task Force on Practice Guidelines (Writing Committee to Revise the 2002 Guidelines for the Management of Patients With Unstable Angina/Non-ST-Elevation Myocardial Infarction) , American College of Emergency Physicians , Society for Cardiovascular Angiography and Interventions , Society of Thoracic Surgeons , American Association of Cardiovascular and Pulmonary Rehabilitation , and Society for Academic Emergency Medicine , ACC/AHA 2007 Guidelines for the Management of Patients With Unstable Angina/Non-ST-Elevation Myocardial Infarction: A Report of the American College of Cardiology/American Heart Association Task Force on Practice Guidelines (Writing Committee to Revise the 2002 Guidelines for the Management of Patients With Unstable Angina/Non-ST-Elevation Myocardial Infarction) Developed in Collaboration With the American College of Emergency Physicians, the Society for Cardiovascular Angiography and Interventions, and the Society of Thoracic Surgeons Endorsed by the American Association of Cardiovascular and Pulmonary Rehabilitation and the Society for Academic Emergency Medicine, Journal of the American College of Cardiology. (2007) 50, no. 7, e1–e157, 10.1016/j.jacc.2007.02.013, 17692738. 17692738

[41] CAPRIE Steering Committee , A Randomised, Blinded, Trial of Clopidogrel Versus Aspirin in Patients at Risk of Ischaemic Events (CAPRIE), Lancet. (1996) 348, no. 9038, 1329–1339, 10.1016/s0140-6736(96)09457-3, 8918275. 8918275

[42] Yusuf S. , Zhao F. , Mehta S. R. , Chrolavicius S. , Tognoni G. , Fox K. K. , and Clopidogrel in Unstable Angina to Prevent Recurrent Events Trial Investigators , Effects of Clopidogrel in Addition to Aspirin in Patients With Acute Coronary Syndromes Without ST-Segment Elevation, New England Journal of Medicine. (2001) 345, no. 7, 494–502, 10.1056/NEJMoa010746, 11519503. 11519503

[43] Diener H. C. , Bogousslavsky J. , Brass L. M. , Cimminiello C. , Csiba L. , Kaste M. , Leys D. , Matias-Guiu J. , and Rupprecht H. J. , Aspirin and Clopidogrel Compared With Clopidogrel Alone After Recent Ischaemic Stroke or Transient Ischaemic Attack in High-Risk Patients (MATCH): Randomised, Double-Blind, Placebo-Controlled Trial, Lancet. (2004) 364, no. 9431, 331–337, 10.1016/S0140-6736(04)16721-4, 15276392. 15276392

[44] Sim D. S. , Jeong M. H. , Kim H. S. , Gwon H. C. , Seung K. B. , Rha S. W. , Chae S. C. , Kim C. J. , Cha K. S. , Park J. S. , Yoon J. H. , and KAMIR-NIH Registry Investigators , Clopidogrel Versus Aspirin After Dual Antiplatelet Therapy in Acute Myocardial Infarction Patients Undergoing Drug-Eluting Stenting, Korean Circulation Journal. (2020) 50, no. 2, 120–129, 10.4070/kcj.2019.0166, 31845550. 31845550 PMC6974667

[45] Gimbel M. , Qaderdan K. , Willemsen L. , Hermanides R. , Bergmeijer T. , de Vrey E. , Heestermans T. , Tjon Joe Gin M. , Waalewijn R. , Hofma S. , den Hartog F. , Jukema W. , von Birgelen C. , Voskuil M. , Kelder J. , Deneer V. , and ten Berg J. , Clopidogrel Versus Ticagrelor or Prasugrel in Patients Aged 70 Years or Older With Non-ST-Elevation Acute Coronary Syndrome (POPular AGE): The Randomised, Open-Label, Non-Inferiority Trial, Lancet. (2020) 395, no. 10233, 1374–1381, 10.1016/S0140-6736(20)30325-1, 32334703. 32334703

[46] Koo B. K. , Kang J. , Park K. W. , Rhee T. M. , Yang H. M. , Won K. B. , Rha S. W. , Bae J. W. , Lee N. H. , Hur S. H. , Yoon J. , Park T. H. , Kim B. S. , Lim S. W. , Cho Y. H. , Jeon D. W. , Kim S. H. , Han J. K. , Shin E. S. , Kim H. S. , Koo B. K. , Kang J. , Park K. W. , Rhee T. M. , Lee H. , Yang H. M. , Won K. B. , Rha S. W. , Bae J. W. , Lee N. H. , Hur S. H. , Yoon J. , Park T. H. , Kim B. S. , Lim S. W. , Cho Y. H. , Jeon D. W. , Kim S. H. , Han J. K. , Shin E. S. , Kim H. S. , Han K. R. , Moon K. W. , Oh S. K. , Kim U. , Rhee M. Y. , Kim D. I. , Kim S. Y. , Lee S. Y. , Lee S. U. , Kim S. W. , Kim S. Y. , Jeon H. K. , Cha K. S. , Jo S. H. , Ryu J. K. , Suh I. W. , Choi H. H. , Woo S. I. , Chae I. H. , Shin W. Y. , Kim D. K. , Oh J. H. , Jeong M. H. , and Kim Y. H. , Aspirin Versus Clopidogrel for Chronic Maintenance Monotherapy After Percutaneous Coronary Intervention (HOST-EXAM): An Investigator-Initiated, Prospective, Randomised, Open-Label, Multicentre Trial, Lancet. (2021) 397, no. 10293, 2487–2496, 10.1016/S0140-6736(21)01063-1, 34010616. 34010616

[47] Kang J. , Park K. W. , Lee H. , Hwang D. , Yang H. M. , Rha S. W. , Bae J. W. , Lee N. H. , Hur S. H. , Han J. K. , and Shin E. S. , Aspirin Versus Clopidogrel for Long-Term Maintenance Monotherapy After Percutaneous Coronary Intervention: The HOST-EXAM Extended Study, Circulation. (2023) 147, no. 2, 108–117, 10.1161/CIRCULATIONAHA.122.062770, 36342475. 36342475

[48] Gurbel P. A. , Bliden K. P. , Butler K. , Tantry U. S. , Gesheff T. , Wei C. , Teng R. , Antonino M. J. , Patil S. B. , Karunakaran A. , and Kereiakes D. J. , Randomized Double-Blind Assessment of the ONSET and OFFSET of the Antiplatelet Effects of Ticagrelor Versus Clopidogrel in Patients With Stable Coronary Artery Disease: The ONSET/OFFSET Study, Circulation. (2009) 120, no. 25, 2577–2585, 10.1161/CIRCULATIONAHA.109.912550, 19923168. 19923168

[49] Wallentin L. , Becker R. C. , Budaj A. , Cannon C. P. , Emanuelsson H. , Held C. , Horrow J. , Husted S. , James S. , Katus H. , Mahaffey K. W. , Scirica B. M. , Skene A. , Steg P. G. , Storey R. F. , and Harrington R. A. , Ticagrelor Versus Clopidogrel in Patients With Acute Coronary Syndromes, New England Journal of Medicine. (2009) 361, no. 11, 1045–1057, 10.1056/NEJMoa0904327, 19717846. 19717846

[50] Bonaca M. P. , Bhatt D. L. , Cohen M. , Steg P. G. , Storey R. F. , Jensen E. C. , Magnani G. , Bansilal S. , Fish M. P. , Im K. , Bengtsson O. , Ophuis T. O. , Budaj A. , Theroux P. , Ruda M. , Hamm C. , Goto S. , Spinar J. , Nicolau J. C. , Kiss R. G. , Murphy S. A. , Wiviott S. D. , Held P. , Braunwald E. , and Sabatine M. S. , Long-Term Use of Ticagrelor in Patients With Prior Myocardial Infarction, New England Journal of Medicine. (2015) 372, no. 19, 1791–1800, 10.1056/NEJMoa1500857, 25773268. 25773268

[51] Steg P. G. , Bhatt D. L. , Simon T. , Fox K. , Mehta S. R. , Harrington R. A. , Held C. , Andersson M. , Himmelmann A. , Ridderstråle W. , Leonsson-Zachrisson M. , Liu Y. , Opolski G. , Zateyshchikov D. , Ge J. , Nicolau J. C. , Corbalán R. , Cornel J. H. , Widimský P. , and Leiter L. A. , Ticagrelor in Patients With Stable Coronary Disease and Diabetes, New England Journal of Medicine. (2019) 381, no. 14, 1309–1320, 10.1056/NEJMoa1908077, 31475798. 31475798

[52] Bhatt D. L. , Steg P. G. , Mehta S. R. , Leiter L. A. , Simon T. , Fox K. , Held C. , Andersson M. , Himmelmann A. , Ridderstråle W. , Chen J. , Song Y. , Diaz R. , Goto S. , James S. K. , Ray K. K. , Parkhomenko A. N. , Kosiborod M. N. , McGuire D. K. , Harrington R. A. , Santos V. , Jain A. , Lendel I. , Russo M. , Haught W. H. , Bouza M. , Gogia H. , Banerjee S. , Kichura G. , Kantaros L. , Padron F. , Passi R. , Stone J. , Pursley M. , D′Urso M. , Gardner T. , Bennett J. , Nour K. , Saini S. , Zhang W. , Kumbhani D. , Thomas D. , Angiolillo D. , Bertolet B. , Roman-Miranda A. , Black R. , Manshadi R. , Vaca C. , Blanco A. , Napoli M. , Brabham D. , Akyea-Djamson A. , Desai P. , Prasada S. , Khaira A. , Forgosh L. , Lieber I. , Umpierrez G. , Singal D. , Londono J. , Fraser N. , Ruiz J. , Vega D. , Rodriguez L. , Brown C. , Syed F. , Aggarwala G. , Eaves W. , Foster M. , Gupta D. , Avino D. , Asfour W. , Tonnessen G. , Zhao X. Q. , Singh N. , Brockmyre A. , Lepor N. , Shammas N. , Blick D. , Hearne S. , Prodafikas J. , Carell E. , Izzo M. , Karim A. , Zakhary B. , Atieh M. , Leichter S. , Meadows C. , Hotchkiss D. , Abu-Fadel M. , Wiseman A. , Bander J. , Shah M. , Banerjee S. , Ganim R. , Sopko K. , Khan M. , Lloret R. , Weirick T. , Mehta R. , Thadani U. , Bhargava A. , Kosiborod M. , Moya J. , Staniloae C. , Guerra Y. D. , Chhabra A. , Kosmicki D. , Shaheen W. , Mohammed A. , Bitters J.′ C. , Pattanayak J. , Javier J. , Srivastava S. , Phillips R. , al-Amin J. , Lillestol M. , Simpson P. , Hazan L. , Amin A. , Shah G. , Korpas D. , Platt B. , Dickert J. , Puente O. , Hiotis L. , Doyle T. , Rajan R. , Meholick A. , Gring C. , Hage-Korban E. , Feldman R. , Colfer H. , Butman S. , Foster M. , Hart T. , Huling R. , Eshaghian S. , Quintana O. , Cheung D. , Handel F. , Rodriguez M. , Suh D. , Gordon P. , Pressman G. , Bauer M. , French W. , Barettella M. , Chatrathi S. , Suresh D. , Goldberg R. , Huth M. , Younis L. , Rahman A. , Mascolo R. , Welch M. , Suneja R. , Smith S. , Shurmur S. , Agaiby J. , Jingo A. , Johnston J. , Beth M. , Vlastaris A. , Kemp S. , Taheri H. , Pereira E. , Deyoung M. , Hawa Z. , Smith R. , Galski T. , Garas S. , Reddy M. , Sharma S. , Hargrove J. , Treasure C. , Emerson R. , Haddad T. , Rohr K. , Levinson L. , Gaona R. , Uretsky B. , Maheshwari H. , Lee D. , Kinnaman S. , Singal R. , Geohas J. , Gigliotti O. , Raisinghani A. , Khurana C. , Hella B. , Kelberman M. , Voyce S. , Singh S. , Lo E. , Singh P. , Goodfellow R. , Fischer S. , Lorraine R. , Turner T. , Shanes J. , Busch R. , Broker R. , Zaniewski M. , Pounds K. , Debs-Perez G. , Ong S. , Frandsen B. , Fullington D. , Jaffrani N. , Khan A. , Lee M. , Pouzar J. , Revtyak G. , Gonzalez J. , Nakhle S. , Murillo A. , Young D. , Makam S. , Syed M. , Woolf K. , Grena P. , Alfata S. , Mahal S. , Hoffman D. , Kizhakekuttu T. , Deering J. , Bhavsar J. , Mikesell S. , Wilson W. , Wilson V. , el S. , Spinale F. , Kannarkat V. , Rao S. , Hanson L. , Bertsch J. , Gonzalez-Ortiz E. , Severino N. , Willis J. , Schock J. , Bakhtari L. , Gazmuri R. , Ansari S. , Hall J. , Mehta A. , Shealy N. , Zarich S. , Singh D. , Vora K. , Andrawis N. , Molter D. , Maron D. , Cardona J. , D′Agostino R. , Arshad T. , Samaan R. , Jones D. , Presser D. , Heath J. , Green S. , Bittar G. , Henry S. , Korn D. , Schmedtje J. , Nadar V. , Graham B. , Labroo A. , Clavijo L. , Roseman H. , Ledesma G. , Rosen R. , Dor I. , Kirby W. , Sutton J. , Eder F. , Iteld B. , Gomez-Cortes J. , Buchbinder M. , Kasper J. , Terrelonge A. , Torres G. , Jagielo T. , Alvarez J. , Handelsman Y. , Guillen M. , Richwine R. , Lewy-Alterbaum L. , Corder C. , Arvind M. , Bolshoun D. , Mikhail M. , Minton S. , Alvarado O. , Abbott J. , Cauthen B. , Welter R. , Mintz R. , Cox J. , Quick A. , Weiss M. , Dy J. , Zebrack J. , Gandelman G. , Hegde V. , Silver M. , DeGregorio M. , Lawson W. , Paa C. , Bortnick A. , Krolick M. , Sotolongo R. , Cheirif J. , Kumar P. , Nadar V. , Jetty P. , Patel A. , Kruk M. , Kobielusz-Gembala I. , Rewerska B. , Madrzejewski A. , Milewski K. , Cygler J. , Petryka-Mazurkiewicz J. , Jastrzebski W. , Korecki J. , Fil W. , Prokopczuk J. , Bochenek A. , Wujkowski M. , Witek R. , Konczakowski P. , Miekus P. , Szczasny M. , Musial W. , Cymerman K. , Lampart J. , Mikosinski J. , Szynal S. , Fares I. , Opolski G. , Mazur S. , Wozakowska-Kaplon B. , Bijata-Bronisz R. , Wierucki L. , Losa B. , Drelich G. , Konieczny M. , Starczewski P. , Pawlowicz L. , Jesionowski P. , Jurowiecki J. , Gniot J. , Czyzycki M. , Stania K. , Kucharczyk-Bauman I. , Busz-Papiez B. , Karczmarczyk A. , Sudnik W. , Koszek A. , Kolodziej P. , Skwarna B. , Jaramillo N. , Jankowski M. , Czochra W. , Kinasz L. , Miklaszewicz B. , Stasinska T. , Pluta W. , Basiak M. , Rusicka T. , Niedbal-Yahfouf I. , Popenda G. , Korzeniak R. , Mirek A. , Mariankowski R. , Wojnowski L. , Korol M. , Baszak J. , Podolec P. , Piesiewicz W. , Zurakowski A. , Luengas C. , Skura M. , Pilecki P. , Majchrzak P. , Krzyzagórska E. , Drozd M. , Kaczmarek B. , Sliwinska T. , Zelazowska K. , Sztembis R. , Landa K. , Matyszczak-Toniak L. , Strojek K. , Piepiorka M. , Malinowski R. , Górska M. , Stolarczyk-Sowa E. , Romanowski L. , Zinka E. , Reszka Z. , Skierkowska J. , Uzunow A. , Laskowska-Derlaga E. , Puntus E. , Kosmacheva E. D. , Koziolova N. , Pavlov P. , Supryadkina T. , Didenko Y. , Kopylov P. , Kazakov A. , Aksentiev S. , Vishneva E. , Repin A. , Smolenskaya O. , Mantserova O. , Khrustalev O. , Privalova E. , Konstantinov V. , Boldueva S. , Ezhov A. , Chernyavsky A. , Kamalov G. , Galyavich A. , Zubeeva G. , Nechaeva G. , Shustov S. , Dzhaiani N. , Treshkur T. , Osokina N. , Panov A. , Shutemova E. , Makukhin V. , Kropotina T. , Tsyba L. , Karpov Y. , Sizova J. M. , Ballyuzek M. , Tarasov N. , Demchenko E. , Barbarash O. , Moiseev V. , Markov V. , Kuznetsov V. , Viktorova I. , Sergienko I. , Ermoshkina L. , Khasanov N. , Khlevchuk T. , Baglikov A. , Shalaev S. , Zonova E. , Reznik E. , Haisheva L. , Morugova T. , Lomakin N. , Vishnevsky A. , Shvarts Y. , Magnitskaya O. , Mikhailusova M. , Pavlysh E. , Libov I. , Zateyschikova A. , Kostenko V. , Edin A. , Khovaeva Y. , Zakharov K. , Stryuk R. , Khirmanov V. , Kanorskiy S. , Yakushin S. , Barabashkina A. , Li H. , Zhao Q. , Zhang J. , Ma J. , He Y. , Luo M. , Zhang A. , Zhang N. , Chai Y. , Ma G. , Wang H. , Liu Z. , He L. , Song Z. , Dong X. , Tao L. , Li Z. , Su X. , Kong X. , Niu H. , Ge J. , Luo Z. , Huang W. , Peng D. , Yuan Z. , Milanova M. , Tenev D. , Gogov A. , Karageorgiev D. , Kolchev T. , Rusev N. , Georgieva N. , Kondov R. , Rusinov V. , Petrov I. , Stanchev G. , Konteva M. , Dincheva A. , Yaneva Z. , Vatova R. , Ilieva K. , Runev N. , Kolomanov B. , Petrov I. , Iliev N. , Tisheva S. , Chompalova B. , Tokmakova M. , Raev D. , Byanov K. , Markov D. , Mihov L. , Mihov A. , Milcheva N. , Minchev M. , Mollov M. , Borisov B. , Tihchev T. , Karakolev V. , Dimov B. , Georgiev S. , Smilov L. , Koo B. K. , Ahn T. , Hong S. J. , Yoon J. , Oh S. K. , Jeong M. H. , Kim D. I. , Chang K. , Kim W. , Hahn J. Y. , Cha K. S. , Lee J. H. , Choi S. W. , Nam C. W. , Chae I. H. , Park Y. H. , Tahk S. J. , Shin W. Y. , Chae J. K. , Kim B. J. , Bae J. W. , Park W. J. , Rha S. W. , Choi Y. J. , Hwang J. Y. , Park H. S. , Baracioli L. , Guimaraes F. , Vasconcellos E. , Saraiva J. , Pereira A. , Santos Q. , Rossi P. , Maia L. , Madeira M. , Pereira M. , Botelho R. , Reis G. , Eliaschewitz F. , Borges J. , Nascimento C. , Fortes J. A. , de Souza W. , Pimentel P. , Hissa M. , Franchetti M. , Precoma D. , Ortiz C. , Hernandes M. , Saporito W. , dos Santos F. R. , Kormann A. , Neuenschwander F. , Dutra O. , Rassi N. , Tanajura L. , Souza J. , Junior D. S. , Leaes P. , Forte A. , Bonansea T. C. , Marin J. , Machado B. , Cerqueira M. J. , Silva F. , Michalaros Y. , Manenti E. , Cercato C. , Figueiredo E. , Liu M. E. , Wang Y. C. , Lee T. M. , Fang C. , Wu Y. W. , Ueng K. C. , Sheu H. H. , Lai W. T. , Hsieh I. C. , Chen Z. C. , Lee M. J. , Chiang C. , Shyu K. G. , Hsia C. H. , Mar G. Y. , Chan S. H. , Wu C. C. , Tseng W. K. , Chang K. C. , Yeh H. I. , Wang J. H. , Hou C. , Sorokina I. , Dolzhenko M. , Horoshko O. , Karpenko O. , Rudenko L. , Vakaliuk I. , Kulyk A. , Levchenko O. , Prokhorov O. , Reshotko D. , Sorokivskyy M. , Velichko N. , Maslovskyi V. , Teliatnikova Z. , Dotsenko S. , Krakhmalova O. , Kraiz I. , Zharinova V. , Bula L. , Kaydashev I. , Molodtsov V. , Rasputina L. , Pidlisna V. , Lysunets O. , Kravchenko A. , Glushko L. , Khomazyuk T. , Svyshchenko Y. , Parkhomenko O. , Mankovsky B. , Abrahamovych O. , Yagensky A. , Stanislavchuk M. , Vasilyeva L. , Sokolova L. , Sychov O. , Tseluyko V. , Kyrychenko I. , Rishko M. , Furkalo S. , Gallo R. , Bertrand O. , Mehta S. , Constance C. , Sussex B. , Zadra R. , Kouz S. , Chehayeb R. , Pandey A. , Dion D. , Bailey G. , Hill L. , Ramanathan K. , Dorsch M. , Nanji A. , Babapulle M. , Montigny M. , Gosselin G. , Dehghani P. , Rupka D. , le May M. , Pichette F. , St-Maurice F. , Teefy P. , Mansour S. , Kassam S. , Cheung S. , Siega A. D. , O′Keefe D. , Sabbah E. , Bell A. , Chouinard G. , Wong B. , Miller M. , Gaudet D. , Lachance P. , Bata I. , Petrella R. , Gossard D. , Dumas R. , Ing D. , Boyrazian H. , Bessoudo R. , Huynh T. T. , Hart R. , Belle-Isle J. , Shukla D. , Kelly A. , Mazza G. , Cha J. , Henein S. , Frechette A. , Vizel S. , Liutkus J. F. , O′Mahony M. , Halperin F. , Kooy J. , Graham J. , Bailey A. , Wojcik R. , Wilderman I. , Turi T. , Motyovszki Á. , Merkely B. , Király C. , Andrássy P. , Sárszegi Z. , Fülöp T. , Zilahi Z. , Édes I. , Papp A. , Müller G. , Czigány A. , Zólyomi S. , Korányi L. , Takács J. , Juhász F. , Benczur B. , Kancz S. , Földi A. , Nagy A. C. , Bakai J. , Greschik I. , Püski L. , Nagy L. , Kirschner R. , Kuchar R. , Hajek P. , Busak L. , Michalik D. , Matyasek I. , Marusincova I. , Kucera D. , Jerabek O. , Honkova M. , Dedek V. , Rihacek I. , Kos P. , Slaby J. , Machkova M. , Zidkova E. , Elbl L. , Grunfeldova H. , Carda J. , Mrozek V. , Maly J. , Milkovic R. , Malecha J. , Skalicka H. , Oral I. , Krcova E. , Lisa L. , Belohlavek J. , Miklik R. , Cermak O. , Bednarova J. , Peroutka Z. , Spinar J. , Wilke A. , Appel K. F. , Taggeselle J. , Förster A. , Toursarkissian N. , Schmidt E. , Bott J. , al-Zoebi A. , Hennig D. , Fischer S. , Schön N. , Sauter J. , Simonis G. , Nischik R. , Rieker W. , Schenkenberger I. , Behnke T. , Klausmann G. , Jeserich M. , Trenk D. , Weigmann I. , Reuter H. , Rummel R. , von Münchhausen C. , von Engelhardt C. , Horibe E. , Shibasaki T. , Sato T. , Kakuta T. , Michishita I. , Tan M. , Ishiki R. , Aoyama T. , Higashiue S. , Niijima Y. , Idogaki A. , Hasegawa T. , Kiyosue A. , Tomobuchi Y. , Kawamitsu K. , Kawasaki S. , Hata Y. , Fukui K. , Seki K. , Takenaka T. , Abe M. , Utsu N. , Oono A. , Mitsuo K. , Sueyoshi A. , Hirohata A. , Tsujimoto M. , Ueda O. , Takase S. , Suzuki M. , Sakuragi S. , Yamamoto F. , Fujimoto N. , Kakinoki S. , Sugiura T. , Sugino H. , Nakamura T. , Goto S. , Kadokami T. , Uehara H. , Ono M. , Yokoya K. , Koike A. , Komatsu S. , Sonoda M. , Ueno H. , Doi T. , Takagi Y. , Fujimoto K. , Eki Y. , Okubo M. , Sasaki K. , van Eck M. , Ronner E. , The S. , van de Wal R. , Nierop P. , de Nooijer C. , The S. , The S. , Werner H. , Westendorp I. , van der Zwaan C. , Crijns H. , Cornel J. H. , Strikwerda S. , Bos R. , de Melker E. , Kuijper A. , Louwerenburg H. , Plomp J. , Dantzig J. M. , Prins F. , van Kesteren H. , Willems F. , Amoroso G. , Carnero G. , Duronto E. , Besada D. , Chacon C. , Zangroniz P. , Solis S. , Liberman A. , Sernia V. , Alvarisqueta A. , Maffei L. , Vilamajo O. G. , García C. , Sicer M. , Muntaner J. , Bordonava A. , Albisu J. , Zanini A. , Rista L. , Hominal M. , Estrada J. N. , Prado A. , Gosparini D. M. , Schiavi B. , Castillo A. G. , Ruíz J. G. , Martinez G. R. , López V. G. , Rosas E. L. , Lopez G. R. , Cantu E. G. , de los Ríos Ibarra M. , Padilla F. P. , Carrasco J. P. , Carrillo L. V. , García J. D. , Askar A. N. , Salinas C. A. , Gamba M. A. , Sanchez C. G. , Cantú A. G. , Sánchez R. V. , Madrigal J. C. , Urbano R. H. , Romo A. Í. , González Juanatey J. R. , Racugno P. , Fillat A. C. , de la Torre Hernández J. M. , Peláez J. A. , Cortada J. B. , Pavia P. G. , Navarro M. J. , Asenjo R. M. , Díaz F. F. , Peligero E. B. , Manterola F. A. , Ortiz A. F. , Mediavilla García J. D. , Ortuño F. M. , Vera T. R. , González A. S. , Viñas J. A. , Fernández Portales F. J. , Mayordomo P. S. , Ojeda F. B. , Domínguez A. R. , González R. S. , Guerrero D. B. , Ruiz Nodar J. M. , Marimon X. G. , Margáez J. G. , Aguilera R. M. , Díaz Fernández J. F. , Zamorano Gómez J. L. , González V. B. , del Blanco B. G. , Pérez I. P. , Moreno M. R. , Ereño A. R. , García Lledó J. A. , Prieto J. , Villablanca A. , Raffo C. , Pincetti C. , Conejeros C. , Roman O. , Rodriguez M. , Varleta P. , Goldberg C. , Sandoval J. , Arriagada G. , Leon L. , Potthoff S. , Cobos J. , Figueroa C. , Makotoko E. , Fourie N. , Burgess L. , Nortje H. , Theron R. , Pillai P. , Ranjith N. , Trokis J. , Pillay S. , Reddy J. , Nunkoo T. , Kapp C. , Urbach D. , Distiller L. , Horak A. , van Zyl L. , Coetzee K. , Punt Z. , Bayat J. , Dawood S. , Mitha I. , Padayachee T. , Hoosen F. , Dalby A. , Prabhavathi , Gowdaiah P. , Mehta V. , Chag M. , Gadkari M. , Ramamurthee K. , das A. , Sawhney J. S. , Banerjee S. , Sathe P. , Adhyapak S. , Nguyen T. , Pham V. , do H. , Nguyen A. , Nguyen H. , Truong B. , Jamil-Copley S. , Lang C. , Pell A. , Zaman A. , Storey R. , Swanson N. , Smith S. , Sharman D. , Braganza D. , Hammond P. , Moriarty A. , Bain S. , Pye M. , Sharp A. , Blagden M. , Randeva H. , Myhill T. , Viswanathan G. , Keeling P. , Clifford P. , Saxena M. , Lyons K. , McMurray J. , Jaafar F. , Murphy C. , Cartwright S. , Abouglila K. , Antalik L. , Krajci P. , Urban M. , Fazekas F. , Pella D. , Koleny D. , Vykoukalova T. , Macek V. , Vinanska D. , Jamriskova L. , Such S. , Fulop P. , Farsky S. , Bugan V. , Strbova J. , Micko K. , Palka Jr J. , Sivak V. , Kristensen D. , Refsgaard J. , Holmvang L. , Dixen U. , Nielsen H. , Egstrup K. , Jensen L. O. , Sykulski R. , Rasmussen O. , Andries A. , Luckow A. , Nielsen G. , Sørensen T. , Wongvipaporn C. , Chamnarnphol N. , Srimahachota S. , Sansanayudh N. , Kuanprasert S. , Tresukosol D. , Sookananchai B. , Kanadasi M. , Ozcan T. , Kucuk M. , Ongen Z. , Okuyan E. , Arat A. , Acikel S. , Yalcin A. , Guray U. , Ceyhan C. , Ozer N. , Arslan S. , Angerås O. , Johnston N. , Weiderman A. C. , Bandh S. , Hansen O. , Larnefeldt H. , Kusiak D. , Lindholm C. J. , Hedman A. , Erlinge D. , Curiac D. , Lundman P. , Zucconi-Mazzini R. , Aladellie L. , Jensen J. , Verwerft J. , Vrolix M. , Faes D. , Striekwold H. , Sinnaeve P. , Timmermans P. , Guedes A. , Delforge M. , Nimmegeers J. , Stammen F. , Buysschaert I. , Hoffer E. , Hollanders G. , Vervoort G. , Coussement P. , de Maeseneire S. , Janssens L. , Gravdal S. A. , Risberg K. , Gullestad L. , Hofseth O. D. , Nilsen D. , Lappegård K. T. , van den Heuvel C. , Gibbs C. , Khusrawi A. , Arora S. , Tomala T. , Kjærnli T. , Berg-Johansen J. , Hagemeier R. , Skjelvan G. , Colquhoun D. , Amerena J. , Morbey C. , Hammett C. , Dart A. , Lehman R. , Hamilton A. , Worthley M. , Purnell P. , Whelan A. , MacIsaac R. , Arya K. , Linjawi S. , Proietto J. , Prasad L. , Rodriguez A. , Godoy A. , Rodriguez V. , Berrospi P. , Chavez C. , Negron S. , Heredia J. , Medina F. , Manrique H. , Cabrera W. , Cordova F. , Quinteros T. , Haro J. M. , Regalado S. , Guitton J. , Arbanil H. , Pansieri M. , Decoulx E. , Goube P. , de Labriolle A. , Labeque J. N. , Range G. , Cottin Y. , Montalescot G. , Cayla G. , Danchin N. , Angoulvant D. , Ferrario E. , Elbaz M. , Dubreuil O. , Steg P. G. , Fontaine C. , Sorbets E. , Omer H. , al-Saif S. , al-Faleh H. , al-Shehri A. , Alshehri H. , Bazari R. , Hei P. , Ying M. , Chan M. , Wong M. , Ma R. , Siu S. C. , Tsang C. C. , Ferrario M. , Assanelli E. , Senni M. , Piatti P. , Calabrò P. , Urbinati S. , Michisanti M. , Varbella F. , de Cosmo S. , Trevisan R. , Bellotti S. , di Pasquale G. , Bongo A. S. , Uguccioni M. , Mannucci E. , Mauro C. , Ragonese M. , Fresco C. , Turturo M. , Marcucci R. , Lievano Triana M. J. , Arana C. , Accini J. , Botero R. , Muzyk-Osikowicz M. , Dada F. T. , Vallejo G. S. , Manzur F. , Isaza D. , Molina D. , Mesa J. G. , Quintero A. , Nyman K. , Mäkelä J. , Strand J. , Nieminen S. , Taurio J. , Kuusela M. , Valle T. , Pietilä M. , Kekki S. , Strandberg T. , Klutstein M. , Greenberg G. , Rozenman Y. , Chorin E. , Roguin A. , Lewis B. , Bashkin A. , Tan E. , Prado J. P. , Ferrolino A. , Babilonia N. , Barbas B. , Matiga G. , Coching R. M. , Drexel H. , Brath H. , Schnack C. , Hanusch U. , Fließer-Görzer E. , Paulweber B. , Ebenbichler C. , Prager R. , Huber K. , Wolzt M. , Auer J. , Berger R. , Schernthaner G. H. , Stanciulescu G. , Creteanu M. , Spiridon M. , Dobreanu V. , Vinereanu D. , Iosipescu L. C. , Istratoaie O. , Coman I. , Militaru C. , Cinteza M. , Bhatt D. L. , Fox K. , Harrington R. A. , Leiter L. A. , Mehta S. R. , Simon T. , Andersson M. , Himmelmann A. , Steg P. G. , Diaz R. , Amerena J. , Huber K. , Sinnaeve P. R. , Nicolau J. C. , Kerr Saraiva J. F. , Petrov I. , Leiter L. A. , Mehta S. R. , Corbalán R. , Ge J. , Zhao Q. , Botero R. , Widimský P. , Kristensen S. D. , Hartikainen J. , Danchin N. , Darius H. , Tse H. F. , Kiss R. G. , Pais P. , Lev E. , de Luca L. , Goto S. , Ramos López G. A. , Cornel J. H. , Kontny F. , Medina F. , Babilonia N. A. , Opolski G. , Vinereanu D. , Zateyshchikov D. A. , Ruda M. , Elamin O. , Kovář F. , Dalby A. J. , Jeong M. H. , Bueno H. , James S. K. , Chiang C. E. , Tresukosol D. , Ongen Z. , Ray K. K. , Parkhomenko A. , McGuire D. K. , Kosiborod M. N. , Nguyen T. Q. , Andersson M. , Chen J. , Himmelmann A. , Leonsson-Zachrisson M. , and Ridderstråle W. , Ticagrelor in Patients With Diabetes and Stable Coronary Artery Disease With a History of Previous Percutaneous Coronary Intervention (THEMIS-PCI): A Phase 3, Placebo-Controlled, Randomised Trial, Lancet. (2019) 394, no. 10204, 1169–1180, 10.1016/S0140-6736(19)31887-2, 31484629. 31484629

[53] Baber U. , Dangas G. , Cohen D. J. , Gibson C. M. , Mehta S. R. , Angiolillo D. J. , Pocock S. J. , Krucoff M. W. , Kastrati A. , Ohman E. M. , Steg P. G. , Badimon J. , Zafar M. U. , Chandrasekhar J. , Sartori S. , Aquino M. , and Mehran R. , Ticagrelor With Aspirin or Alone in High-Risk Patients After Coronary Intervention: Rationale and Design of the TWILIGHT Study, American Heart Journal. (2016) 182, 125–134, 10.1016/j.ahj.2016.09.006, 27914492. 27914492

[54] Vranckx P. , Valgimigli M. , Jüni P. , Hamm C. , Steg P. G. , Heg D. , van Es G. , McFadden E. , Onuma Y. , van Meijeren C. , Chichareon P. , Benit E. , Möllmann H. , Janssens L. , Ferrario M. , Moschovitis A. , Zurakowski A. , Dominici M. , van Geuns R. , Huber K. , Slagboom T. , Serruys P. W. , Windecker S. , and GLOBAL LEADERS Investigators , Ticagrelor Plus Aspirin for 1 Month, Followed by Ticagrelor Monotherapy for 23 Months vs Aspirin Plus Clopidogrel or Ticagrelor for 12 Months, Followed by Aspirin Monotherapy for 12 Months After Implantation of a Drug-Eluting Stent: A Multicentre, Open-Label, Randomised Superiority Trial, Lancet. (2018) 392, no. 10151, 940–949, 10.1016/S0140-6736(18)31858-0, 30166073. 30166073

[55] Kim B. K. , Hong S. J. , Cho Y. H. , Yun K. H. , Kim Y. H. , Suh Y. , Cho J. Y. , Her A. Y. , Cho S. , Jeon D. W. , Yoo S. Y. , and TICO Investigators , Effect of Ticagrelor Monotherapy vs Ticagrelor With Aspirin on Major Bleeding and Cardiovascular Events in Patients With Acute Coronary Syndrome: The TICO Randomized Clinical Trial, Jama. (2020) 323, no. 23, 2407–2416, 10.1001/jama.2020.7580, 32543684. 32543684 PMC7298605

[56] Zhao Q. , Zhu Y. , Xu Z. , Cheng Z. , Mei J. , Chen X. , and Wang X. , Effect of Ticagrelor Plus Aspirin, Ticagrelor Alone, or Aspirin Alone on Saphenous Vein Graft Patency 1 Year After Coronary Artery Bypass Grafting: A Randomized Clinical Trial, Jama. (2018) 319, no. 16, 1677–1686, 10.1001/jama.2018.3197, 29710164. 29710164 PMC5933396

[57] Wiviott S. D. , Braunwald E. , McCabe C. H. , Montalescot G. , Ruzyllo W. , Gottlieb S. , Neumann F. J. , Ardissino D. , de Servi S. , Murphy S. A. , Riesmeyer J. , Weerakkody G. , Gibson C. M. , and Antman E. M. , Prasugrel Versus Clopidogrel in Patients With Acute Coronary Syndromes, New England Journal of Medicine. (2007) 357, no. 20, 2001–2015, 10.1056/NEJMoa0706482, 17982182. 17982182

[58] Capodanno D. , Milluzzo R. P. , and Angiolillo D. J. , Intravenous Antiplatelet Therapies (Glycoprotein IIb/IIIa Receptor Inhibitors and Cangrelor) in Percutaneous Coronary Intervention: From Pharmacology to Indications for Clinical Use, Therapeutic Advances in Cardiovascular Disease. (2019) 13, 1753944719893274, 10.1177/1753944719893274, 31823688. 31823688 PMC6906352

[59] Levine G. N. , Bates E. R. , Bittl J. A. , Brindis R. G. , Fihn S. D. , Fleisher L. A. , Granger C. B. , Lange R. A. , Mack M. J. , Mauri L. , and Mehran R. , 2016 ACC/AHA Guideline Focused Update on Duration of Dual Antiplatelet Therapy in Patients With Coronary Artery Disease: A Report of the American College of Cardiology/American Heart Association Task Force on Clinical Practice Guidelines: An Update of the 2011 ACCF/AHA/SCAI Guideline for Percutaneous Coronary Intervention, 2011 ACCF/AHA Guideline for Coronary Artery Bypass Graft Surgery, 2012 ACC/AHA/ACP/AATS/PCNA/SCAI/STS Guideline for the Diagnosis and Management of Patients With Stable Ischemic Heart Disease, 2013 ACCF/AHA Guideline for the Management of ST-Elevation Myocardial Infarction, 2014 AHA/ACC Guideline for the Management of Patients With Non-ST-Elevation Acute Coronary Syndromes, and 2014 ACC/AHA Guideline on Perioperative Cardiovascular Evaluation and Management of Patients Undergoing Noncardiac Surgery, Circulation. (2016) 134, no. 10, e123–e155, 10.1161/CIR.0000000000000404, 27026020. 27026020

[60] Valgimigli M. , Bueno H. , Byrne R. A. , Collet J. P. , Costa F. , Jeppsson A. , Jüni P. , Kastrati A. , Kolh P. , Mauri L. , Montalescot G. , and ESC Scientific Document Group; ESC Committee for Practice Guidelines (CPG); ESC National Cardiac Societies , 2017 ESC Focused Update on Dual Antiplatelet Therapy in Coronary Artery Disease Developed in Collaboration With EACTS: The Task Force for Dual Antiplatelet Therapy in Coronary Artery Disease of the European Society of Cardiology (ESC) and of the European Association for Cardio-Thoracic Surgery (EACTS), European Heart Journal. (2018) 39, no. 3, 213–260, 10.1093/eurheartj/ehx419, 28886622. 28886622

[61] Eikelboom J. W. , Connolly S. J. , Bosch J. , Dagenais G. R. , Hart R. G. , Shestakovska O. , Diaz R. , Alings M. , Lonn E. M. , Anand S. S. , Widimsky P. , Hori M. , Avezum A. , Piegas L. S. , Branch K. R. H. , Probstfield J. , Bhatt D. L. , Zhu J. , Liang Y. , Maggioni A. P. , Lopez-Jaramillo P. , O’Donnell M. , Kakkar A. K. , Fox K. A. A. , Parkhomenko A. N. , Ertl G. , Störk S. , Keltai M. , Ryden L. , Pogosova N. , Dans A. L. , Lanas F. , Commerford P. J. , Torp-Pedersen C. , Guzik T. J. , Verhamme P. B. , Vinereanu D. , Kim J. H. , Tonkin A. M. , Lewis B. S. , Felix C. , Yusoff K. , Steg P. G. , Metsarinne K. P. , Cook Bruns N. , Misselwitz F. , Chen E. , Leong D. , and Yusuf S. , Rivaroxaban With or Without Aspirin in Stable Cardiovascular Disease, New England Journal of Medicine. (2017) 377, no. 14, 1319–1330, 10.1056/NEJMoa1709118, 28844192. 28844192

[62] Mauri L. , Kereiakes D. J. , Yeh R. W. , Driscoll-Shempp P. , Cutlip D. E. , Steg P. G. , Normand S. L. , Braunwald E. , Wiviott S. D. , Cohen D. J. , Holmes D. R. , and DAPT Study Investigators , Twelve or 30 Months of Dual Antiplatelet Therapy After Drug-Eluting Stents, New England Journal of Medicine. (2014) 371, no. 23, 2155–2166, 10.1056/NEJMoa1409312, 25399658. 25399658 PMC4481318

[63] Hahn J. Y. , Song Y. B. , Oh J. H. , Chun W. J. , Park Y. H. , Jang W. J. , Im E. S. , Jeong J. O. , Cho B. R. , Oh S. K. , Yun K. H. , and SMART-CHOICE Investigators , Effect of P2Y12 Inhibitor Monotherapy vs Dual Antiplatelet Therapy on Cardiovascular Events in Patients Undergoing Percutaneous Coronary Intervention: The SMART-CHOICE Randomized Clinical Trial, JAMA. (2019) 321, no. 24, 2428–2437, 10.1001/jama.2019.8146, 31237645. 31237645 PMC6593635

[64] Natsuaki M. , Morimoto T. , Yamamoto E. , Shiomi H. , Furukawa Y. , Abe M. , Nakao K. , Ishikawa T. , Kawai K. , Yunoki K. , and Shimizu S. , One-Year Outcome of a Prospective Trial Stopping Dual Antiplatelet Therapy at 3 Months After Everolimus-Eluting Cobalt-Chromium Stent Implantation: ShortT and OPtimal Duration of Dual AntiPlatelet Therapy After Everolimus-Eluting Cobalt-Chromium Stent (STOPDAPT) Trial, Cardiovascular Intervention and Therapeutics. (2016) 31, no. 3, 196–209, 10.1007/s12928-015-0366-9, 26518420. 26518420 PMC4923071

[65] Watanabe H. , Domei T. , Morimoto T. , Natsuaki M. , Shiomi H. , Toyota T. , Ohya M. , Suwa S. , Takagi K. , Nanasato M. , Hata Y. , and STOPDAPT-2 Investigators , Effect of 1-Month Dual Antiplatelet Therapy Followed by Clopidogrel vs 12-Month Dual Antiplatelet Therapy on Cardiovascular and Bleeding Events in Patients Receiving PCI: The STOPDAPT-2 Randomized Clinical Trial, JAMA. (2019) 321, no. 24, 2414–2427, 10.1001/jama.2019.8145, 31237644. 31237644 PMC6593641

[66] Costa F. , Van Klaveren D. , Feres F. , James S. , Räber L. , Pilgrim T. , Hong M. K. , Kim H. S. , Colombo A. , Steg P. G. , and Bhatt D. L. , Dual Antiplatelet Therapy Duration Based on Ischemic and Bleeding Risks After Coronary Stenting, Journal of the American College of Cardiology. (2019) 73, no. 7, 741–754, 10.1016/j.jacc.2018.11.048, 30784667. 30784667

[67] Tanik V. O. , Cinar T. , Arugaslan E. , Karabag Y. , Hayiroglu M. I. , Cagdas M. , Rencuzogullari I. , and Uluganyan M. , The Predictive Value of PRECISE-DAPT Score for In-Hospital Mortality in Patients With ST-Elevation Myocardial Infarction Undergoing Primary Percutaneous Coronary Intervention, Angiology. (2019) 70, no. 5, 440–447, 10.1177/0003319718807057, 30322265. 30322265

[68] Liu D. , Xu W. P. , Xu H. , Zhao L. , and Jin D. Q. , Efficacy and Safety of Clopidogrel Versus Aspirin Monotherapy for Secondary Prevention in Patients With Coronary Artery Disease: A Meta-Analysis, Frontiers in Cardiovascular Medicine. (2023) 10, 1265983, 10.3389/fcvm.2023.1265983, 37915738. 37915738 PMC10616297

[69] Li S. , Wang D. , Han X. , Zhang D. , Deng H. , and Pan G. , Antiplatelet Strategy for Patients With Acute Coronary Syndrome and Chronic Kidney Disease: A Systematic Review and Meta-Analysis, Frontiers in Cardiovascular Medicine. (2025) 12, 1527667, 10.3389/fcvm.2025.1527667, 40051435. 40051435 PMC11882542

[70] De Filippo O. , D′Ascenzo F. , and De Ferrari G. M. , Antiplatelet Therapy in Acute Coronary Syndrome Patients With Impaired Renal Function, JACC Cardiovascular Interventions. (2021) 14, no. 17, 1867–1869, 10.1016/j.jcin.2021.07.026, 34446391. 34446391

[71] Su X. , Yan B. , Wang L. , Lv J. , Cheng H. , and Chen Y. , Effect of Antiplatelet Therapy on Cardiovascular and Kidney Outcomes in Patients With Chronic Kidney Disease: A Systematic Review and Meta-Analysis, BMC Nephrology. (2019) 20, no. 1, 10.1186/s12882-019-1499-3, 31390997. PMC6686545 31390997

[72] Segal R. , Lubart E. , Leibovitz A. , Berkovitch M. , Habot B. , Yaron M. , and Caspi D. , Early and Late Effects of Low-Dose Aspirin on Renal Function in Elderly Patients, American Journal of Medicine. (2003) 115, no. 6, 462–466, 10.1016/s0002-9343(03)00436-4, 14563503. 14563503

[73] Capranzano P. and Angiolillo D. J. , Antithrombotic Management of Elderly Patients With Coronary Artery Disease, JACC Cardiovascular Interventions. (2021) 14, no. 7, 723–738, 10.1016/j.jcin.2021.01.040, 33826494. 33826494

[74] Szummer K. , Montez-Rath M. E. , Alfredsson J. , Erlinge D. , Lindahl B. , Hofmann R. , Ravn-Fischer A. , Svensson P. , and Jernberg T. , Comparison Between Ticagrelor and Clopidogrel in Elderly Patients With an Acute Coronary Syndrome: Insights From the SWEDEHEART Registry, Circulation. (2020) 142, no. 18, 1700–1708, 10.1161/CIRCULATIONAHA.120.050645, 32867508. 32867508

[75] Roe M. T. , Goodman S. G. , Ohman E. M. , Stevens S. R. , Hochman J. S. , Gottlieb S. , Martinez F. , Dalby A. J. , Boden W. E. , White H. D. , Prabhakaran D. , Winters K. J. , Aylward P. E. , Bassand J. P. , McGuire D. K. , Ardissino D. , Fox K. A. A. , and Armstrong P. W. , Elderly Patients With Acute Coronary Syndromes Managed Without Revascularization, Circulation. (2013) 128, no. 8, 823–833, 10.1161/CIRCULATIONAHA.113.002303, 23852610. 23852610

[76] De Luca G. , Verdoia M. , Savonitto S. , Piatti L. , Grosseto D. , Morici N. , Bossi I. , Sganzerla P. , Tortorella G. , Cacucci M. , and Murena E. , Elderly ACS 2 Investigators. Impact of Diabetes on Clinical Outcome Among Elderly Patients With Acute Coronary Syndrome Treated With Percutaneous Coronary Intervention: Insights From the ELDERLY ACS 2 Trial, Journal of Cardiovascular Medicine. (2020) 21, no. 6, 453–459, 10.2459/JCM.0000000000000978, 32355067. 32355067

[77] Schüpke S. , Neumann F. J. , Menichelli M. , Mayer K. , Bernlochner I. , Wöhrle J. , Richardt G. , Liebetrau C. , Witzenbichler B. , Antoniucci D. , Akin I. , Bott-Flügel L. , Fischer M. , Landmesser U. , Katus H. A. , Sibbing D. , Seyfarth M. , Janisch M. , Boncompagni D. , Hilz R. , Rottbauer W. , Okrojek R. , Möllmann H. , Hochholzer W. , Migliorini A. , Cassese S. , Mollo P. , Xhepa E. , Kufner S. , Strehle A. , Leggewie S. , Allali A. , Ndrepepa G. , Schühlen H. , Angiolillo D. J. , Hamm C. W. , Hapfelmeier A. , Tölg R. , Trenk D. , Schunkert H. , Laugwitz K. L. , and Kastrati A. , Ticagrelor or Prasugrel in Patients With Acute Coronary Syndromes, New England Journal of Medicine. (2019) 381, no. 16, 1524–1534, 10.1056/NEJMoa1908973, 31475799. 31475799

[78] Valgimigli M. , Frigoli E. , Heg D. , Tijssen J. , Jüni P. , Vranckx P. , Ozaki Y. , Morice M. C. , Chevalier B. , Onuma Y. , Windecker S. , Tonino P. A. L. , Roffi M. , Lesiak M. , Mahfoud F. , Bartunek J. , Hildick-Smith D. , Colombo A. , Stanković G. , Iñiguez A. , Schultz C. , Kornowski R. , Ong P. J. L. , Alasnag M. , Rodriguez A. E. , Moschovitis A. , Laanmets P. , Donahue M. , Leonardi S. , and Smits P. C. , Dual Antiplatelet Therapy After PCI in Patients at High Bleeding Risk, New England Journal of Medicine. (2021) 385, no. 18, 1643–1655, 10.1056/NEJMoa2108749, 34449185. 34449185

[79] De Servi S. , Landi A. , Savonitto S. , Morici N. , De Luca L. , Montalto C. , Crimi G. , De Rosa R. , and De Luca G. , Antiplatelet Strategies for Older Patients With Acute Coronary Syndromes: Finding Directions in a Low-Evidence Field, Journal of Clinical Medicine. (2023) 12, no. 5, 10.3390/jcm12052082, 36902869. PMC10003933 36902869

[80] De Rosa R. , Piscione F. , Galasso G. , De Servi S. , and Savonitto S. , Antiplatelet Therapy in Very Elderly and Comorbid Patients With Acute Coronary Syndromes, Journal of Geriatric Cardiology. (2019) 16, no. 2, 103–113, 10.11909/j.issn.1671-5411.2019.02.006, 30923541. 30923541 PMC6431602

[81] Kalenderoğlu K. , Çınar T. , and Hayıroğlu M. İ. , What Is the Optimal Antiplatelet Therapy in Type 2 Diabetes Mellitus Patients With Small Diameter Stents?, Anatolian Journal of Cardiology. (2025) 29, no. 8, 448–449, 10.14744/AnatolJCardiol.2025.5347, 40418167. 40418167 PMC12336708

[82] Fischer Q. , Pham V. , Seret G. , Brami P. , Picard F. , and Varenne O. , Antiplatelet Therapy for Treatment of Coronary Artery Disease in Older Patients, Archives of Cardiovascular Diseases. (2024) 117, no. 6-7, 441–449, 10.1016/j.acvd.2024.02.008, 38658313. 38658313

